# Structural design and dynamic characteristics analysis of braided composite two-stage gear transmission system

**DOI:** 10.1038/s41598-024-56411-9

**Published:** 2024-03-07

**Authors:** Weiliang Zhang

**Affiliations:** Institute of Mechanical Engineering, Baoji University of Arts and Sciences, Baoji, 721016 China

**Keywords:** Braided composite, Mixed metal gear, Dynamic characteristic analysis, Vibration resonance, Materials science, Structural materials, Composites

## Abstract

In order to realize the lightweight design of the transmission system, the braided composite material is applied to the two-stage gear transmission system. According to the structural characteristics of the two-stage gear reducer box, the whole box is designed to be assembled with the braided base and the box wall. Woven composite materials are applied to the web parts of mixed metal composite gears to realize the design goal of lightweight gears. Then, under the assumption of ignoring the influence of friction, bearings and other factors on the system, the dynamic model of the two-stage gear transmission system considering the box is established. By normalizing and dimensionless processing of the equations, the dimensionless differential equations of motion are obtained. The fourth-order Runge–Kutta method is used to analyze the relationship between the connection parameters and the dynamic characteristics of the system under the two working conditions of rigid and flexible connection between the composite base and the box wall.Through the analytical analysis of vibration displacement of two-stage gear reducer and box, the theoretical basis is found for the numerical analysis results. Finally, the dynamic characteristics of the transmission system are studied by vibration resonance analysis through high and low frequency interference. It is found that in a certain frequency range, with the decrease of the mass and moment of inertia of the transmission parts corresponding to the mixed metal composite gear, the amplitude-frequency characteristic Q of the lightweight gear and gearbox transmission system is slightly lower than that of the common gear and gearbox system, and the stability of the system is increased, and the dynamic characteristics of the system are improved.

## Introduction

Three-dimensional braided composites are widely used in aviation, aerospace, navigation, transportation and medical treatment. Compared with traditional materials, three-dimensional braided composites have the advantages of light weight, good toughness, strong fatigue and corrosion resistance, and designable material parameters and properties^1–5^.

In recent years, the research on composite gear mainly focuses on lightweight, parametric design of braided composite gear and mechanical properties research. Shadi et al.^6^ used a spring-damper element with variable stiffness to simulate the meshing process of gears, and studied the dynamic influence of mass reduction on paired meshing spur gears. Mijiyawa et al.^7^ studied the application of polypropylene/wood composite in gears and its influence on the elastic modulus and tensile strength of gears. Mohsenzadeh and TAVČAR et al.^8,9^ studied the wear, thermal behavior and failure mechanism of composite gears under different friction coefficients by means of gear test-bed and finite element analysis. Roberts et al.^10^ used braided composite materials instead of metal materials to design the gear center web, and found that the hybrid gear can reduce weight by 20% without sacrificing strength. Liu Fengfeng et al.^11^ carried out parametric design of three-dimensional five-way braided composite gear, and analyzed the contact and bending stress of composite gear by ABAQUS software. Catera et al.^12^ established a prediction model of natural frequency of mixed metal-composite gear by using multi-scale method considering yarn interweaving, cross-sectional geometry, volume fraction and local fiber orientation, and the results showed that the accuracy was significantly improved compared with the model that regarded the material as completely isotropic. Waller et al.^13^ found that the steel-composite gear made by combining the steel teeth with the fiber-reinforced polymer composite core significantly reduced the weight without affecting the gear performance. Compared with the research on the structure and performance of braided composite transmission system, the research on the dynamic characteristics of gear reducer is more mature and extensive^14^. Wang Shuguo et al.^15^ used lumped mass method to study the nonlinear vibration bifurcation characteristics of multi-gap two-stage gear system under the comprehensive influence of various nonlinear factors. Rao et al.^16^ studied the nonlinear dynamic characteristics and fault coupling characteristics of gear pair system by numerical method. Yang Fuchun et al.^17^ established an 8-degree-of-freedom nonlinear dynamic model of two-stage spur gear reducer with multiple backlash.

However, at present, in the research of 3D braided composites, there is a lack of systematic theoretical research on the application of composites to box parts. Many research work focuses on a single part of mechanical system, and there is no systematic in-depth research on the application of composites to the whole transmission system such as gears and reducer boxes. In addition, there is a lack of research on composite transmission system from the perspective of dynamics and transmission system, taking into account factors such as connection parameters, gear quality, input excitation and interference.

On the basis of the above research, this paper mainly designs the structure of two-stage gear reducer box and mixed metal composite gear, and analyzes the dynamic characteristics of two-stage gear transmission system. The thesis is divided into seven parts. In the second section, the box of braided composite two-stage gear reducer and mixed metal composite gear are designed. In "Structure design and system dynamics model of braided composite two-stage gear transmission system", the dynamic model of composite two-stage gear transmission system is established. In the fourth section, the relationship between the connection parameters and the dynamic characteristics of the system is numerically analyzed under the two working conditions of rigid and flexible connection between the composite base and the box wall. In "Analytical analysis of vibration displacement of two-stage gear reducer and box", the vibration displacement of two-stage gear reducer and box is analyzed analytically, so as to find a theoretical basis for the numerical analysis results. In "The vibration resonance analysis of two-stage gear reducer and box.", high and low frequency interference are applied to the box respectively, and the dynamic characteristics of the system are studied by vibration resonance analysis of the system. In "Conclusion", we state the main conclusions.

## Structural design of braided composite two-stage gear transmission system

### Structural design of braided composite two-stage gear reducer box

**Figure 1:**
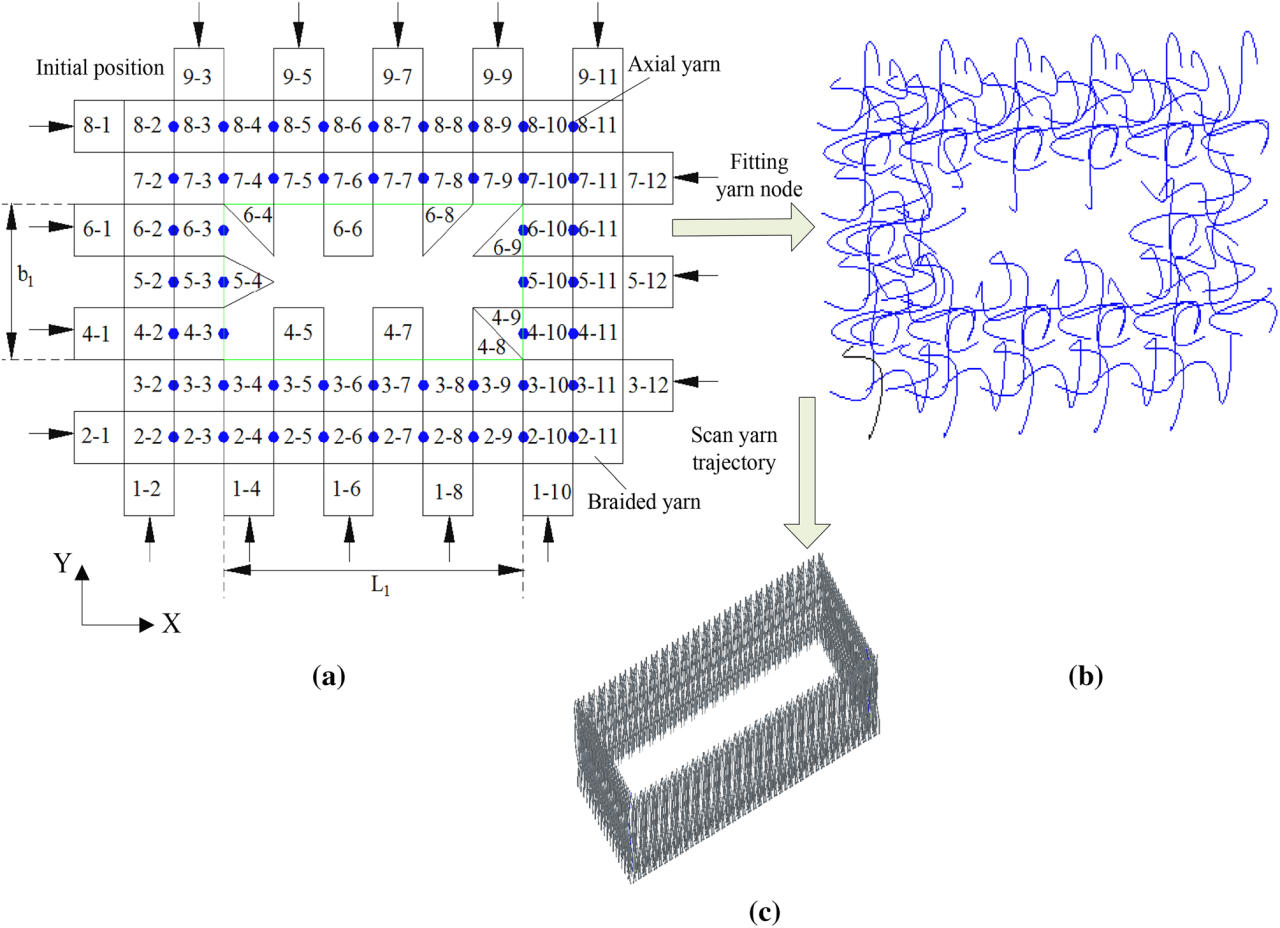
The braiding process of 3D five-way box wall. **(a)** Braiding initial position. **(b)** Yarn trajectory. **(c)** Composite preform model.

In Fig. 1a,● is the axial yarn carrier, which only moves in the X direction and does not move in the Y direction during braiding. In Fig. 1a, there are ten braided yarns in the X-direction main body part, N = 10, and seven braided yarns in the Y-direction main body part, M = 7^2–4^.

**Figure 2:**
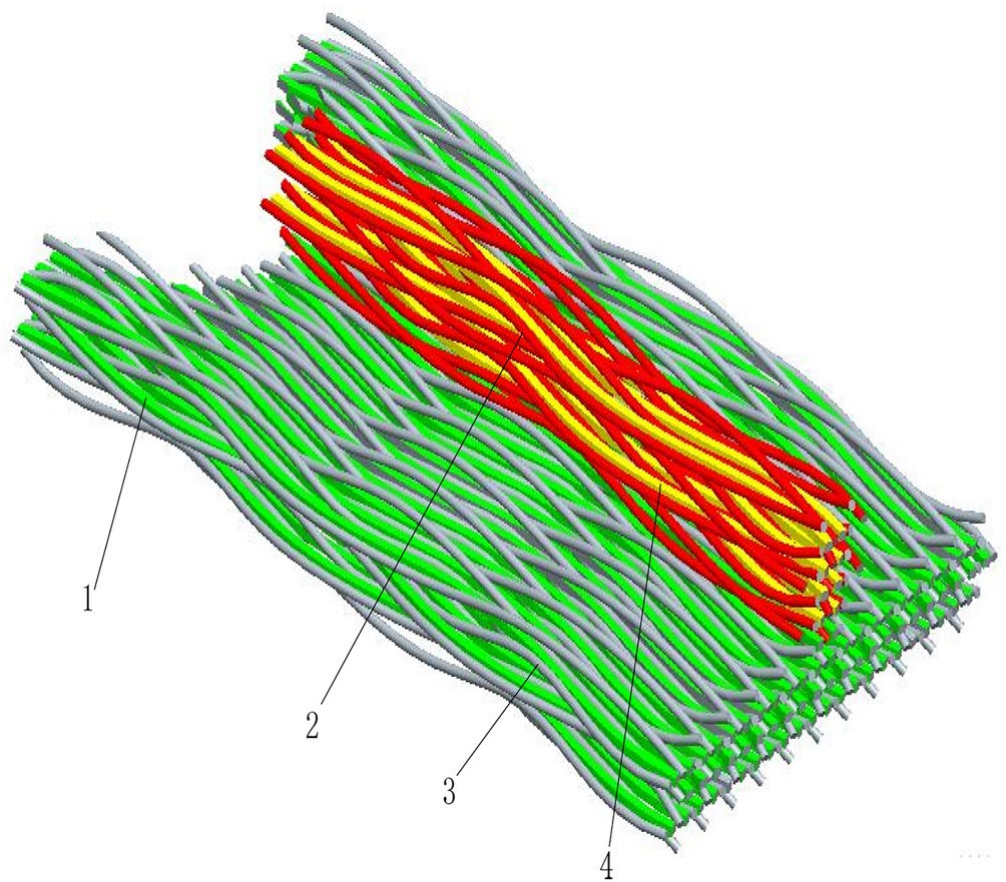
Local braiding model of 3D five-way box reinforcement. (1) Box shaft yarn, (2) ribbed braiding yarn, (3) box braiding yarn, (4) ribbed shaft yarn.

Figure 1 shows the braiding principle of the three-dimensional five-way box wall (3D five-way braiding means that the fibers in the mesoscopic model have five different directions, that is, four directions for braiding yarns and one direction for axial yarns in Fig. 1).Taking the yarn position shown in Fig. 1 as the initial state, the yarn can be moved in four steps(in Fig. 1, the yarn is woven in X and Y directions, with four steps and one cycle) to obtain the yarn trajectory of the woven box wall as shown in Fig. 1b, and the three-dimensional solid model of the box wall can be obtained by scanning the yarn trajectory, as shown in Fig. 1c, which is a braided composite box wall preform. In the figure, $b_{1}$ is the width of the inner wall of the woven box, and $L_{1}$ is the length of the inner wall of the woven box. Because the outer wall of the gear transmission system generally needs to be designed with reinforcing ribs, it is necessary to design the weaving of the reinforcing ribs on the basis of the weaving of the box wall in Fig. 1, so as to ensure that the box wall and the reinforcing ribs are woven and formed at one time. Figure 2 is a three-dimensional solid model of three-dimensional five-way box rib local weaving. In Fig. 2, one is box axial yarn (green part in the figure), two is rib woven yarn (red part in the figure), three is box woven yarn (silver part in the figure) and four is rib axial yarn (yellow part in the figure)^5^.

The woven box wall shown in Fig. 1 and the T-shaped woven reinforcing rib shown in Fig. 2 are superposed to make a three-dimensional five-way composite box yarn preform model, and then the yarn preform and the matrix are compounded, compacted and solidified to make the three-dimensional five-way composite box. In the process of three-dimensional modeling of woven composite box, we mainly use the above braiding principle to get the yarn trajectory, then scan the trajectory in the software to get the three-dimensional solid model of the woven box yarn, and then get the three-dimensional solid model of the woven box through Boolean operation with the matrix model^19^.

### Structural design of mixed metal composite gear

**Figure 3:**
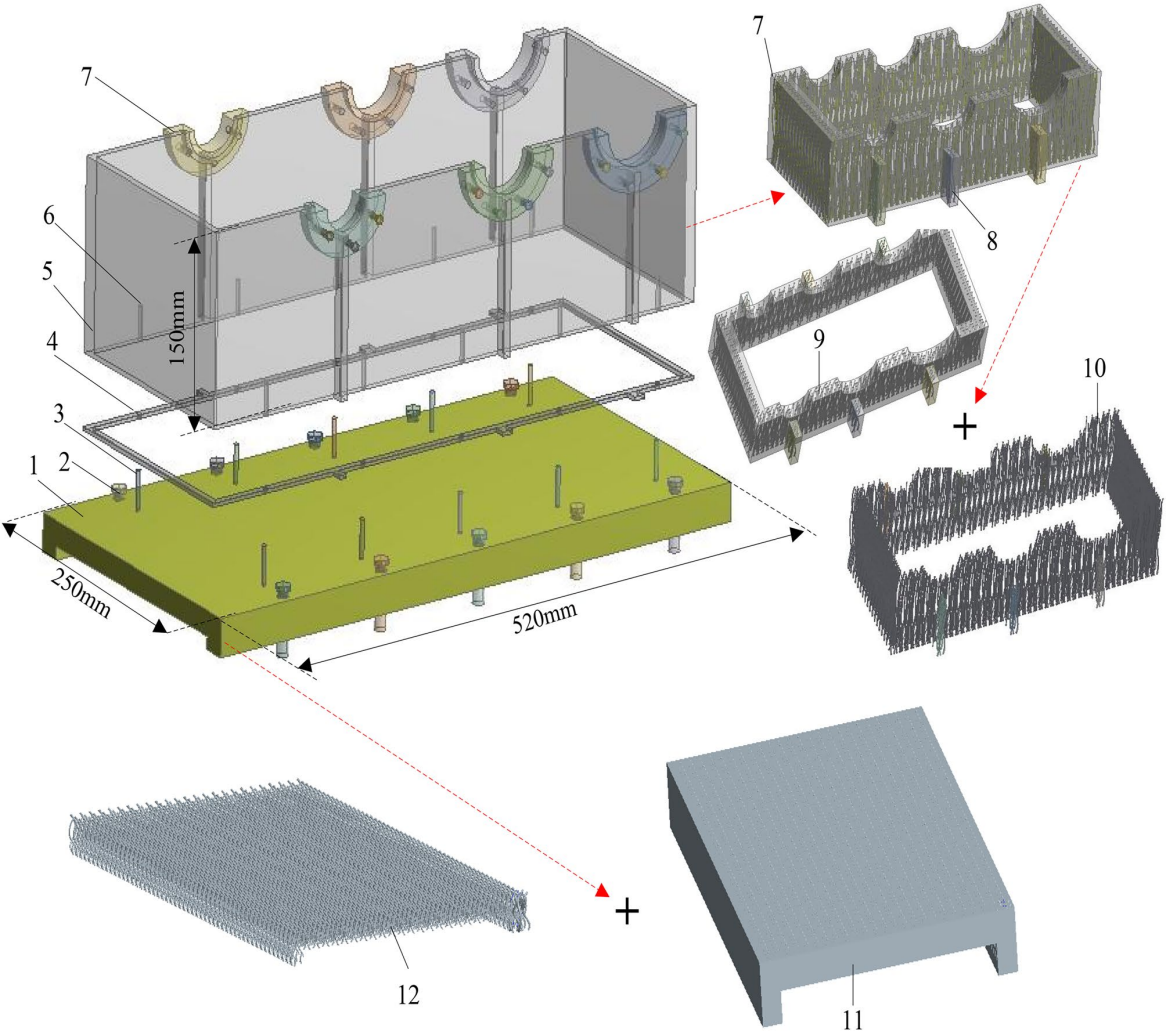
The 3D model of braided composite assembled box. (1) Composite base, (2) fixing bolt for base and frame, (3) fixing bolt for base and composite box, (4) sealing ring, (5) composite box, (6) threaded hole, (7) shaft sleeve, (8) reinforcing rib, (9) composite box base, (10) composite box woven preform yarn, (11) composite base base base, (12) composite base woven preform yarn.

The three-dimensional braided composite box model established in this paper is shown in Fig. 3, which adopts the structure of splicing the braided base and the box wall to realize the design of the composite box. As shown in Fig. 3, the composite base 1 and the box wall 5 are connected by fixing bolts 3. In order to prevent oil leakage at the splicing position, a sealing ring is designed between the base and the box (4 parts in Fig. 3). In addition, considering the wear resistance and the service life of the box, a shaft sleeve 7 is designed at the contact position between the box and the bearing in Fig. 3. The shaft sleeve is made of ordinary structural steel and fixed on the woven box wall 5 by bolts.

**Figure 4:**
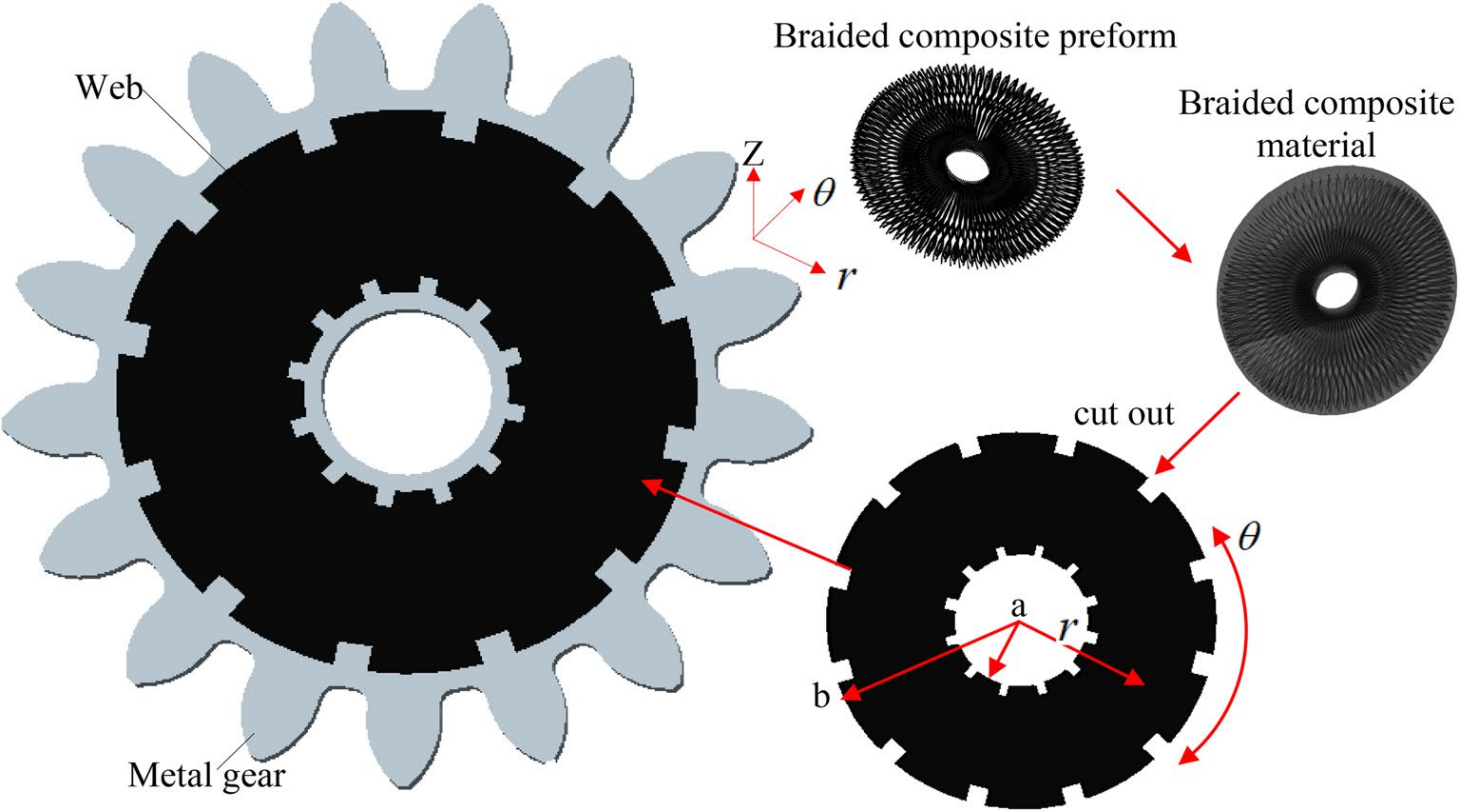
Preparation process of mixed metal composite gear.

The braided composite material is applied to the web parts of mixed metal composite gear, and the preparation process of mixed metal composite gear is shown in Fig. 4. The teeth of the gear are made of metal, and the web of the gear is made of circular transverse braided composite material Take the modulus m = 2.5, the number of teeth Z = 17, the diameter of the indexing circle 42.5 mm, the outside of the composite web 35 mm and the tooth width B = 15 mm as shown in Fig. 4. By comparison, it can be seen that the weight of the mixed metal composite gear can be reduced by 39.25% compared with the traditional metal gear. Therefore, the design shown in Fig. 4 not only meets the design goal of excellent rigidity of the gear as a whole, but also realizes light weight^10–13^.

## Structure design and system dynamics model of braided composite two-stage gear transmission system

**Figure 5:**
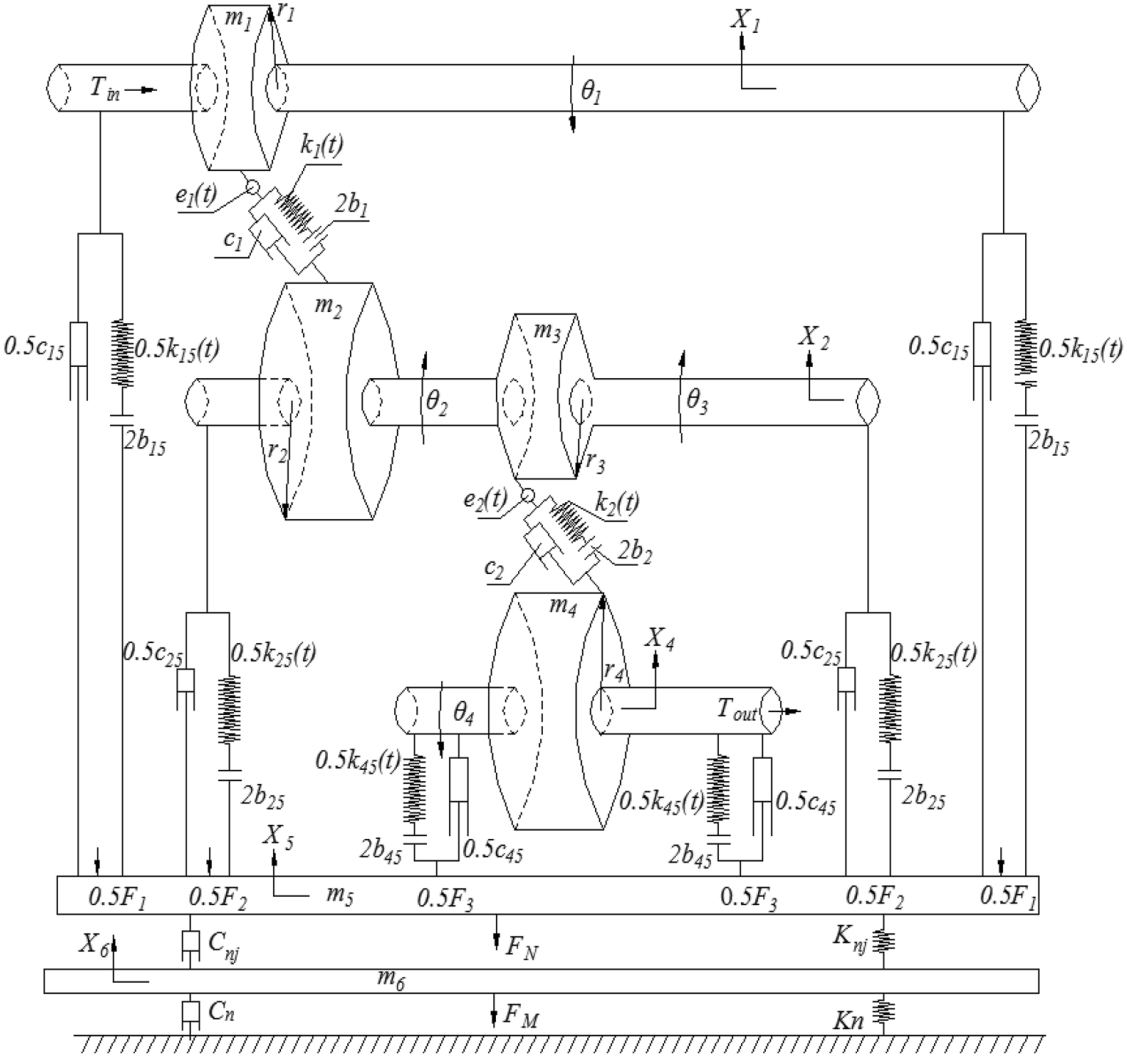
Dynamic model of two-stage gear reducer and box in vertical direction.

The dynamic model of composite two-stage gear reducer is shown in Fig. 5. In Fig. 5,$m_{i}$ is the mass of the gear respectively.$r_{i}$ is the radius of the indexing circle of the gear respectively.$\theta_{i}$ is the angle of gear rotation.$x_{i}$ is the vertical displacement of the shaft.$K_{n}$ is the contact stiffness between the box and the ground in the vertical direction.$C_{n}$ is the vertical contact damping between the box and the ground.$c_{i5}$ is contact damping in vertical direction.$k_{i5} (t)$ is the contact stiffness in vertical direction.$c_{1}$ and $c_{2}$ are damping between meshing gears.$k_{1} (t)$ and $k_{2} (t)$ are the time-varying meshing stiffness between meshing gears.$e_{1} (t)$ and $e_{2} (t)$ comprehensive transmission errors between meshing gears.$F_{i}$ is the force that the bearing acts on the box.$b_{i}$ is the backlash of meshing gear.$b_{i5}$ is the clearance between the bearing and the box. $F_{1} = c_{15} (\dot{x}_{1} - \dot{x}_{5} ) + k_{15} (t)\xi_{1}$, $F_{2} = c_{25} (\dot{x}_{2} - \dot{x}_{5} ) + k_{25} (t)\xi_{2}$, $F_{3} = c_{45} (\dot{x}_{4} - \dot{x}_{5} ) + k_{45} (t)\xi_{3}$^14–18^.

According to the corresponding relationship between different parameters in Fig. 5, ignoring the influence of friction, bearing and other factors in the transmission system, the kinematics equations of the two-stage gear transmission system with box are established, as shown in Formula (1)^18,20^.

1 $$\left\{ {\begin{array}{*{20}c} {m_{1} \left( {\ddot{x}_{1} - \ddot{x}_{5} } \right) + c_{15} (\dot{x}_{1} - \dot{x}_{5} ) + k_{15} (t)\xi_{1} {\text{ = cos}}\alpha \left( {k_{1} (t)\kappa_{1} { + }c_{1} (\dot{x}_{1} - \dot{x}_{2} )} \right)} \\ \begin{gathered} \hfill m_{23} \left( {\ddot{x}_{2} - \ddot{x}_{5} } \right) + c_{25} (\dot{x}_{2} - \dot{x}_{5} ) + k_{25} (t)\xi_{2} + {\text{cos}}\alpha (k_{2} (t)\kappa_{2} \\ \hfill { + }c_{2} (\dot{x}_{2} - \dot{x}_{4} )) = {\text{cos}}\alpha \left( {k_{1} (t)\kappa_{1} { + }c_{1} (\dot{x}_{1} - \dot{x}_{2} )} \right) \\ \end{gathered} \\ {m_{4} \left( {\ddot{x}_{4} - \ddot{x}_{5} } \right) + c_{45} (\dot{x}_{4} - \dot{x}_{5} ) + k_{45} (t)\xi_{3} {\text{ = cos}}\alpha {(}k_{2} (t)\kappa_{2} { + }c_{2} (\dot{x}_{2} - \dot{x}_{4} ))} \\ {F_{1} { = }c_{15} (\dot{x}_{1} - \dot{x}_{5} ) + k_{15} (t)\xi_{1} } \\ {F_{2} { = }c_{25} (\dot{x}_{2} - \dot{x}_{5} ) + k_{25} (t)\xi_{2} } \\ {F_{3} { = }c_{45} (\dot{x}_{4} - \dot{x}_{5} ) + k_{45} (t)\xi_{3} } \\ {F{ = }F_{1} { + }F_{2} { + }F_{3} } \\ {m_{5} \ddot{x}_{5} + C_{n} \dot{x}_{5} + K_{n} x_{5} = F} \\ \end{array} } \right.$$

In formula (1), the values and definitions of parameters are detailed in reference^20^. Simplify formula (1) and define dimensionless time and displacement scale, $\tau = w_{h} t$, $\overline{x} (t) = x(t)/b$.The Eq. (1) is normalized and dimensionless, and the dimensionless differential equations of motion are obtained.

2 $$\left\{ {\begin{array}{*{20}c} \begin{gathered} \hfill \ddot{x}_{1} { = }\frac{{{\text{cos}}\alpha \left( {k_{1} (t)\kappa_{1} { + }c_{1} (\dot{x}_{1} - \dot{x}_{2} )} \right) - c_{15} (\dot{x}_{1} - \dot{x}_{5} ) - k_{15} (t)\xi_{1} }}{{m_{1} }} + \frac{{c_{15} (\dot{x}_{1} - \dot{x}_{5} ) + k_{15} (t)\xi_{1} }}{{m_{5} }}{ + } \\ \hfill \frac{{c_{25} (\dot{x}_{2} - \dot{x}_{5} )}}{{m_{5} }}{ + }\frac{{k_{25} (t)\xi_{2} + c_{45} (\dot{x}_{4} - \dot{x}_{5} ) + k_{45} (t)\xi_{3} - C_{nj} (\dot{x}_{5} - \dot{x}_{6} ) - K_{nj} (x_{5} - x_{6} )}}{{m_{5} }} \\ \end{gathered} \\ \begin{gathered} \hfill \ddot{x}_{2} = \frac{{{\text{cos}}\alpha \left( {k_{1} (t)\kappa_{1} { + }c_{1} (\dot{x}_{1} - \dot{x}_{2} )} \right) - {\text{cos}}\alpha (k_{2} (t)\kappa_{2} + c_{2} (\dot{x}_{2} - \dot{x}_{4} )) - k_{25} (t)\xi_{2} - c_{25} (\dot{x}_{2} - \dot{x}_{5} )}}{{m_{23} }} + \frac{{c_{15} (\dot{x}_{1} - \dot{x}_{5} )}}{{m_{5} }} \\ \hfill \frac{{k_{15} (t)\xi_{1} }}{{m_{5} }}{ + }\frac{{c_{25} (\dot{x}_{2} - \dot{x}_{5} )}}{{m_{5} }}{ + }\frac{{k_{25} (t)\xi_{2} + c_{45} (\dot{x}_{4} - \dot{x}_{5} ) + k_{45} (t)\xi_{3} - C_{nj} (\dot{x}_{5} - \dot{x}_{6} ) - K_{nj} (x_{5} - x_{6} )}}{{m_{5} }} \\ \end{gathered} \\ \begin{gathered} \hfill \ddot{x}_{4} { = }\frac{{{\text{cos}}\alpha {(}k_{2} (t)\kappa_{2} { + }c_{2} (\dot{x}_{2} - \dot{x}_{4} )) - c_{45} (\dot{x}_{4} - \dot{x}_{5} ) - k_{45} (t)\xi_{3} }}{{m_{4} }} + \frac{{c_{15} (\dot{x}_{1} - \dot{x}_{5} ) + k_{15} (t)\xi_{1} }}{{m_{5} }} \\ \hfill \frac{{c_{25} (\dot{x}_{2} - \dot{x}_{5} )}}{{m_{5} }} + \frac{{k_{25} (t)\xi_{2} + c_{45} (\dot{x}_{4} - \dot{x}_{5} ) + k_{45} (t)\xi_{3} - C_{nj} (\dot{x}_{5} - \dot{x}_{6} ) - K_{nj} (x_{5} - x_{6} )}}{{m_{5} }} \\ \end{gathered} \\ {\ddot{x}_{5} = \frac{{c_{15} (\dot{x}_{1} - \dot{x}_{5} ) + k_{15} (t)\xi_{1} + c_{25} (\dot{x}_{2} - \dot{x}_{5} ) + k_{25} (t)\xi_{2} + c_{45} (\dot{x}_{4} - \dot{x}_{5} ) + k_{45} (t)\xi_{3} - C_{nj} (\dot{x}_{5} - \dot{x}_{6} ) - K_{nj} (x_{5} - x_{6} )}}{{m_{5} }}} \\ {\ddot{x}_{6} = \frac{{c_{15} (\dot{x}_{1} - \dot{x}_{5} ) + k_{15} (t)\xi_{1} + c_{25} (\dot{x}_{2} - \dot{x}_{5} ) + k_{25} (t)\xi_{2} + c_{45} (\dot{x}_{4} - \dot{x}_{5} ) + k_{45} (t)\xi_{3} - C_{n} \dot{x}_{6} - K_{n} x_{6} }}{{m_{6} }}} \\ \end{array} } \right.$$

## Numerical analysis of dynamic characteristics of composite two-stage gear reducer and box

### Analysis of flexible connection system between braided base and box wall

If the pre-tightening force between the base and the box wall is too small, the threaded connection is not tight enough, or the stiffness of the sealing ring at the joint is insufficient, the structural connection parameters of the base and the box wall will have a certain impact on the dynamic characteristics of the whole system.

Take the mass of mixed metal composite gear m_1_ = 0.0224 kg, m_2_ = 0.8576 kg, m_3_ = 0.06144 kg, m_4_ = 0.6208 kg, and the mass parameters of ordinary gear are selected according to Table 1.Using the simulation parameters shown in Table 1 and the fourth-order Runge–Kutta method shown in Eq. (3), the differential Eq. (2) is numerically analyzed.

**Table 1:** The parameters of common gear.

Mass (kg)	Rotary inertia (kg m^2^)	Module	Gear tooth number	Gear width (mm)
m_1_ = 0.035	J_1_ = 0.0025	1.5	Z_1_ = 35	30
m_2_ = 1.34	J_2_ = 0.6368	1.5	Z_2_ = 140	30
m_3_ = 0.096	J_3_ = 0.0067	1.5	Z_3_ = 45	25
m_4_ = 0.97	J_4_ = 0.4304	1.5	Z_4_ = 130	25

In Eq. (2), let $$y_{i} = \overline{x}_{i} ,z_{i} = \overline{{\dot{x}}}_{i} ,$$$$X = \left( {\begin{array}{*{20}c} {y_{i} } \\ {z_{i} } \\ \end{array} } \right) = \left( {\begin{array}{*{20}c} {\overline{x}_{i} } \\ {\overline{{\dot{x}}}_{i} } \\ \end{array} } \right)$$.If the second derivative is reduced to the first, then: $$\dot{X} = \left( {\begin{array}{*{20}c} {\dot{y}_{i} } \\ {\dot{z}_{i} } \\ \end{array} } \right) = \left( {\begin{array}{*{20}c} {\overline{{\dot{x}}}_{i} } \\ {\overline{{\ddot{x}}}_{i} } \\ \end{array} } \right)$$, the initial conditions are $$X_{0} = \left( {\begin{array}{*{20}c} {\overline{x}_{i} \left( 0 \right)} \\ {\overline{{\dot{x}}}_{i} \left( 0 \right)} \\ \end{array} } \right){ = }\left( {\begin{array}{*{20}c} 0 \\ 0 \\ \end{array} } \right)$$^18,20^.

3 $$\begin{gathered} k_{1} = f(t(i),y(i),z(i)) \hfill \\ L_{1} = g(t(i),y(i),z(i)) \hfill \\ k_{2} = f(t(i) + h/2,y(i) + h/2 \times k_{1} ,z(i) + h/2 \times L_{1} ) \hfill \\ L_{2} = g(t(i) + h/2,y(i) + h/2 \times k_{1} ,z(i) + h/2 \times L_{1} ) \hfill \\ k_{3} = f(t(i) + h/2,y(i) + h/2 \times k_{2} ,z(i) + h/2 \times L_{2} ) \hfill \\ L_{3} = g(t(i) + h/2,y(i) + h/2 \times k_{2} ,z(i) + h/2 \times L_{2} ) \hfill \\ k_{4} = f(t(i) + h,y(i) + h \times k_{3} ,z(i) + h \times L_{3} ) \hfill \\ L_{4} = g(t(i) + h,y(i) + h \times k_{3} ,z(i) + h \times L_{3} ) \hfill \\ y(i + 1) = y(i) + h/6 \times (k_{1} + 2 \times k_{2} + 2 \times k_{3} + k_{4} ) \hfill \\ z(i + 1) = z(i) + h/6 \times (L_{1} + 2 \times L_{2} + 2 \times L_{3} + k_{4} ) \hfill \\ \end{gathered}$$

**Figure 6:**
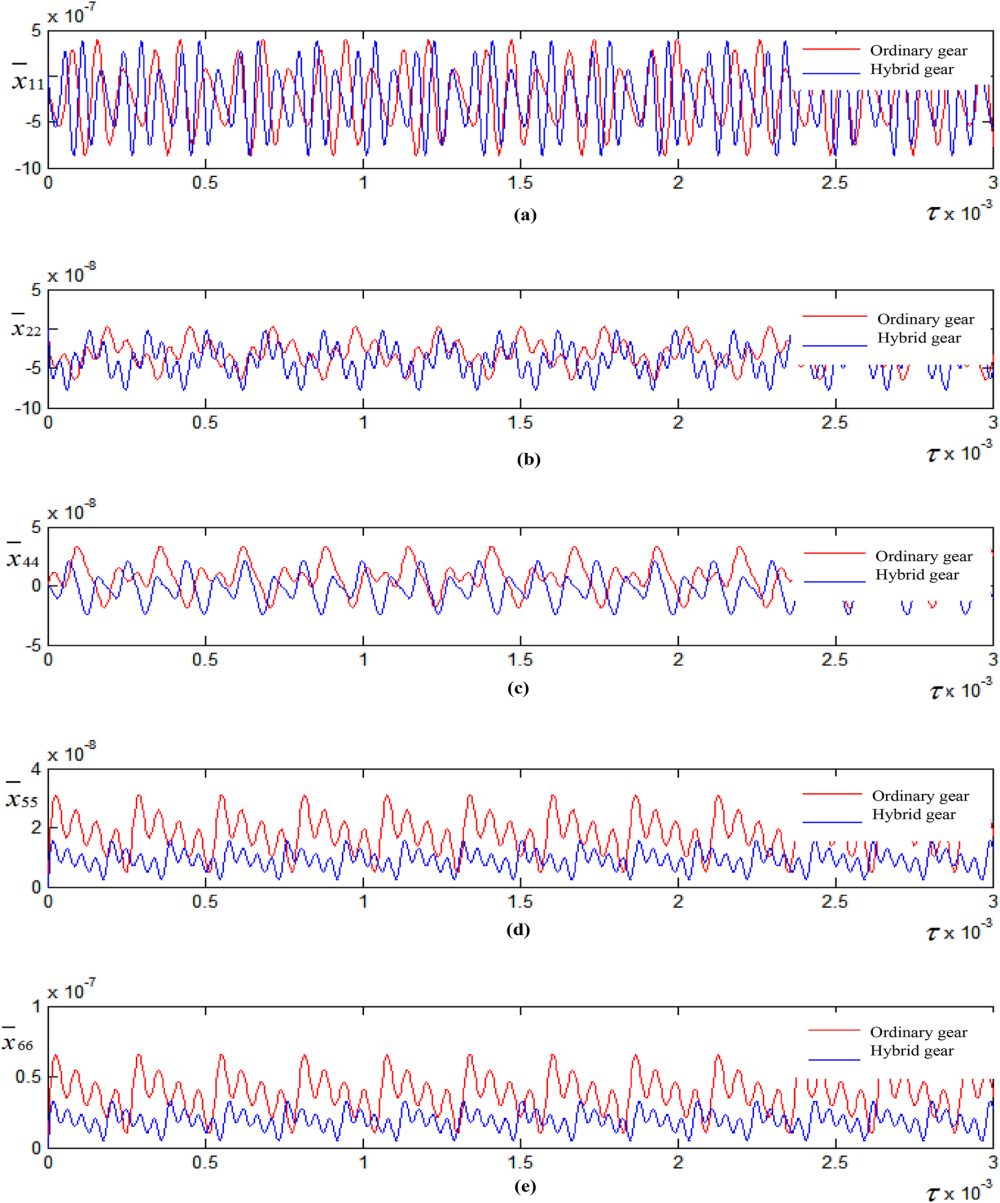
Influence curve of gear weight on flexible connection vibration displacement. (**a**) Vibration displacement curve of shaft 1 in vertical direction; (**b**) vibration displacement curve of shaft 2 in vertical direction; (**c**) vibration displacement curve of shaft 4 in vertical direction; (**d**) vibration displacement curve of box 5 in vertical direction; (**e**) vibration displacement curve of base 6 in vertical direction.

Figure 6 shows the influence curve of gear mass on vibration displacement of flexible connection system. Take the gear mass of metal mixed composite material m_1_ = 0.0224 kg, m_2_ = 0.8576 kg, m_3_ = 0.06144 kg, m_4_ = 0.6208 kg, and the quality parameters of ordinary gear are selected according to Table 1. As can be seen from the figure, with the decrease of the mass of the metal hybrid composite gear, the rotational inertia of the gear decreases, and then the dimensionless meshing frequency of the system decreases, resulting in the change of the time scale of the vibration displacement curves of the two types of gears in Fig. 6a–d, and the delay of the peak and valley of the vibration displacement curves. In addition, as can be seen from Figs. 6a¸b, with the decrease of the mass and moment of inertia of the metal mixed composite gear, the peak and valley slightly decrease, but the periodic relationship remains unchanged. As can be seen from Fig. 6c, the vibration displacement amplitude of the shaft 4 decreases obviously with the decrease of the mass and moment of inertia of the metal mixed composite gear. As can be seen from Fig. 6d, e, the vibration displacement amplitude of the box 5 and the base 6 decreases with the decrease of the gear mass and moment of inertia of the metal mixed composite material, the peak maximum value decreases greatly, the peak-valley minimum value decreases slightly, and the amplitude of the overall curve fluctuation decreases greatly. This conclusion shows that the reduction of the gear mass and moment of inertia in the transmission system is beneficial to the reduction of the vibration amplitude of the supporting parts of the whole system.

### Analysis of rigid connection system between braided base and box wall

In actual normal work, the pre-tightening force of the threaded connection between the base and the box wall is generally large enough, so the contact between them is close enough, so the influence of the threaded connection between the woven base and the box wall on the dynamic characteristics of the composite box can be ignored. At this time, the structure assembled by the base and the box wall can be regarded as a whole, that is, the connection between the base and the box wall is considered as a rigid connection. According to the condition of rigid connection: $x_{5} = x_{6}$, $\dot{x}_{5} = \dot{x}_{6}$, $\ddot{x}_{5} = \ddot{x}_{6}$, $m_{56} = m_{5} + m_{6}$, the last two equations in formula (2) are superimposed, normalized and dimensionless.

**Figure 7:**
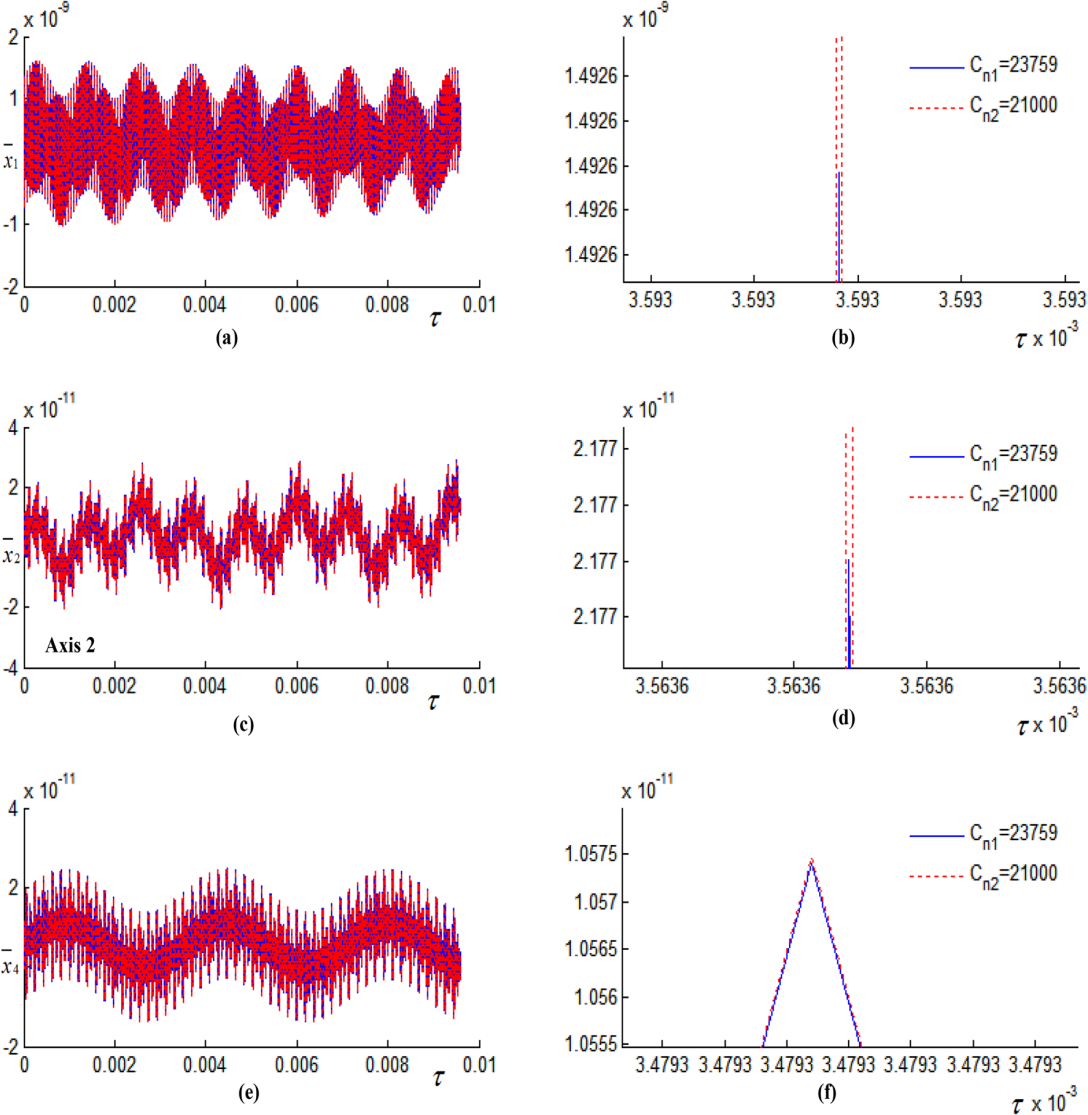
Influence of damping on vibration displacement. (**a**) Vibration displacement curve of shaft 1 in vertical direction; (**b**) the partial enlarged view corresponding to (**a**); (**c**) vibration displacement curve of shaft 2 in vertical direction; (**d**) the partial enlarged view corresponding to (**c**); (**e**) vibration displacement curve of shaft 4 in vertical direction; (**f**) the partial enlarged view corresponding to (**e**).

Take the vertical contact damping between the chassis and the frame of different sizes, and study the influence of damping on the vibration displacement of the system, as shown in Fig. 7. Figure 7b, d, f are partial views corresponding to Fig. 7a, c, e respectively. As can be seen from Fig. 7b, d, with the decrease of contact damping, the vibration displacement amplitude of shaft 1 and shaft 2 increases, but the increase amplitude is small. As can be seen from Fig. 7f, the vibration displacement amplitude of shaft 4 in low-speed stage increases with the decrease of contact damping, and the increase amplitude is slightly larger.

## Analytical analysis of vibration displacement of two-stage gear reducer and box

According to Eq. (2), since the excitation signals are simple harmonic excitations with different frequencies and phases, assuming that the initial phase of the excitation signals is equal to zero, the vibration displacement equation of the box 5 can be written as^21^:

4 $$\ddot{x}_{5} + \frac{{C_{n} \dot{x}_{5} }}{{m_{56} }} + \frac{{K_{n} x_{5} }}{{m_{56} }} = \frac{{F_{ji} }}{{m_{56} }}$$

In the formula (4),

$F_{ji} = F_{cji} + F_{kji}$,$F_{cji} = c_{15} (\dot{x}_{1} - \dot{x}_{2} ) + c_{25} (\dot{x}_{2} - \dot{x}_{5} ) + c_{45} (\dot{x}_{4} - \dot{x}_{5} )$,$F_{kji} = k_{15} (t)\xi_{1} + k_{25} (t)\xi_{2} + k_{45} (t)\xi_{3}$,$F_{ji}$ is the excitation signal.$F_{cji}$ is damping force.$F_{kji}$ is elastic force.Assuming that the vibration displacement of the box 5 is the superposition of harmonic excitations with different frequencies and phases, Eq. (4) can be written as follows:

5 $$\ddot{x}_{5} + \frac{{C_{n} \dot{x}_{5} }}{{m_{56} }} + \frac{{K_{n} x_{5} }}{{m_{56} }} = \frac{{\sum\limits_{i = 1}^{n} {F_{\max }^{i} \sin (\omega_{i} t)} }}{{m_{56} }}$$

In the formula (5), $F_{\max }^{i}$ is the $i$ amplitude of the excitation signal. $\omega_{i}$ is the $i$ frequency of the excitation signal. $n$ is the frequency number of the excitation signal.Then: $B_{s}^{i} = \frac{{F_{\max }^{i} }}{{K_{n} }}$ is the displacement caused by static force $F_{\max }^{i}$.$\varsigma = \frac{{C_{n} }}{{2\sqrt {m_{56} K_{n} } }}$ is viscous damping ratio. $\omega_{n} = \sqrt {\frac{{K_{n} }}{{m_{56} }}}$ is natural angular frequency of undamped system.Accordingly, Formula (5) can be rewritten as:

6 $$\ddot{x}_{5} + 2\varsigma \omega_{n} \dot{x}_{5} + \omega_{n}^{2} x_{5} = B_{s}^{i} \omega_{i}^{2} \sum\limits_{i = 1}^{n} {\sin (\omega_{i} t)}$$

Formula (6) is a second-order linear non-homogeneous differential equation with constant coefficients. According to the theory of ordinary differential equations, the full solution of the differential equation of formula (6) is the general solution of the corresponding homogeneous equation $x^{\prime}_{5}$ and special solution of nonhomogeneous equation $x^{\prime\prime}_{5}$ two parts are superimposed.The general solution of the homogeneous equation corresponding to formula (6) can be written as^22^.

7 $$x^{\prime}_{5} = \sum\limits_{i = 1}^{i = n} {e^{{ - \varsigma \omega_{n} t}} [C_{1i} \cos (\omega_{i} t) + C_{2i} \sin (\omega_{i} t)]}$$

Obviously, in the bracket of formula (7) , $C_{1i} \cos (\omega_{i} t) + C_{2i} \sin (\omega_{i} t) \le C_{1i} + C_{2i}$ with the increase of time, the exponential term of the general solution $e^{{ - \varsigma \omega_{n} t}} \to 0$.Therefore, the general solution is a physical motion that decays with time, and it is a transient response of the system, indicating the free vibration response of the damped system. It is meaningful only for a short period of time after the vibration starts. With the increase of time, the amplitude of the general solution will decay to zero, and the greater the damping, the faster the amplitude of the general solution will decay.

According to $\omega_{n} { = }\sqrt {\frac{{K_{n} }}{{m_{5} }}}$,It can be seen that the natural frequencies of each order of composite box are higher than those of ordinary box in different degrees. Therefore, the decrease of the mass of composite box leads to the increase of viscous damping ratio to a certain extent, which leads to the exponential term of formula (7) $\varsigma \omega_{n}$ enlarge, therefore, the decay process of transient response is accelerated and the decay time is shortened, and the dynamic characteristics are improved.

Since the excitation signal is a simple harmonic signal, the form of the special solution of the non-homogeneous equation in formula (6) can be assumed as follows:

8 $$x^{\prime\prime}_{5} = \sum\limits_{i = 1}^{n} {X_{\max }^{i} \sin (\omega_{i} t - \psi_{c}^{i} )}$$

In the formula (8), $X_{\max }^{i}$ is the amplitude of forced vibration. $\psi_{c}^{i}$ is the phase difference between the displacement response and the excitation signal. Obviously, the excitation signal and the displacement response have the same frequency. The formula (8) is brought into the formula (6) to obtain:

9 $$\sum\limits_{i = 1}^{n} {X_{\max }^{i} \left\{ {(\omega_{n}^{2} - \omega_{i}^{2} )\sin (\omega_{i} t - \psi_{c}^{i} ) + 2\varsigma \omega_{n} \left. {\omega_{i} \cos (\omega_{i} t - \psi_{c}^{i} )} \right\}} \right.} = \sum\limits_{i = 1}^{n} {[B_{s}^{i} \omega_{i}^{2} \sin (\omega_{i} t)]}$$

Expand the excitation signal function on the right side of the equal sign of formula (9) into the form of trigonometric function, and get^21^:

10 $$\begin{array}{*{20}c} {\sum\limits_{i = 1}^{n} {B_{s}^{i} \omega_{i}^{2} \sin (\omega_{i} t)} = \sum\limits_{i = 1}^{n} {\left\{ {\left. {B_{s}^{i} \omega_{i}^{2} \sin (\omega_{i} t - \psi_{c}^{i} + \psi_{c}^{i} )} \right\}} \right.} } \\ { = \sum\limits_{i = 1}^{n} {\left\{ {\left. {B_{s}^{i} \omega_{i}^{2} [\cos \psi_{c}^{i} \sin (\omega_{i} t - \psi_{c}^{i} ) + \sin \psi_{c}^{i} \cos (\omega_{i} t - \psi_{c}^{i} )]} \right\}} \right.} } \\ \end{array}$$

Comparing Eqs. (9) and (10), we get:

11 $$\left\{ {\begin{array}{*{20}c} {\sum\limits_{i = 1}^{n} {\left\{ {\left. {(\omega_{n}^{2} - \omega_{i}^{2} )X_{\max }^{i} } \right\}} \right.} = \sum\limits_{i = 1}^{n} {\left\{ {\left. {B_{s}^{i} \omega_{i}^{2} \cos \psi_{c}^{i} } \right\}} \right.} } \\ {\sum\limits_{i = 1}^{n} {\left\{ {\left. {2\varsigma \omega_{n} \omega_{i} X_{\max }^{i} } \right\}} \right.} = \sum\limits_{i = 1}^{n} {\left\{ {\left. {B_{s}^{i} \omega_{i}^{2} \sin \psi_{c}^{i} } \right\}} \right.} } \\ \end{array} } \right.$$

After the transformation of formula (11), it is brought into $\sin^{2} \psi_{c}^{i} + \cos^{2} \psi_{c}^{i} = 1$,the formula for calculating the phase difference between the amplitude and displacement response of forced vibration lagging behind the excitation signal is obtained:

12 $$\left\{ {\begin{array}{*{20}c} {X_{\max } = \sum\limits_{i = 1}^{n} {\left\{ {\left. {\frac{{B_{s}^{i} \omega_{n}^{2} }}{{\sqrt {(\omega_{n}^{2} - \omega_{i}^{2} )^{2} + (2\varsigma \omega_{n} \omega_{i} )^{2} } }}} \right\}} \right.} } \\ {\psi_{c} = \sum\limits_{i = 1}^{n} {\left\{ {\left. {\arctan \left( {\frac{{\sin \psi_{c}^{i} }}{{\cos \psi_{c}^{i} }}} \right)} \right\} = \sum\limits_{i = 1}^{n} {\left\{ {\left. {\arctan \left( {\frac{{2\varsigma \omega_{n} \omega_{i} }}{{\omega_{n}^{2} - \omega_{i}^{2} }}} \right)} \right\}} \right.} } \right.} } \\ \end{array} } \right.$$

In the formula (12),$X_{\max }$ is the amplitude of forced vibration under the joint action of n excitation signals. $\psi_{c}$ is n excitation signals, and the phase difference between the displacement response and the excitation signals.

Since the natural frequencies of the composite box are higher than those of the ordinary box in different degrees, according to Formula (12), the natural frequencies in the molecules of the formula for calculating the amplitude of the forced vibration show a square relationship, so that the amplitude of the forced vibration of the composite box is higher than that of the ordinary box under the action of the excitation signal. In addition, the increase of the natural frequencies of the composite box can, to a certain extent, lead to the decrease of the phase difference in Eq. (12) and the decrease of the system delay, thus improving the dynamic characteristics of the system.

## The vibration resonance analysis of two-stage gear reducer and box

Because of the accuracy of the contact position between the gearbox and the frame, the manufacturing defects of the gearbox itself, the vibration of the frame, the accuracy error of the rolling elements of the bearings and the eccentricity of the shaft, the actual excitation of the gearbox in the working process is more complicated.

In this paper, the influence of all the above factors on the system is regarded as imposing high and low frequency interference on the input box and taking the contact stiffness frequency between the shaft and the box $\omega_{15} = 0.2$, $\omega_{45} = 0.1$.The vibration resonance analysis of two-stage gear reducer and box system is carried out to study the dynamic characteristics of the system.

The definition formula (13) defines the response amplitude gain Q of the system output at the low frequency signal, which is used to study the degree of vibration resonance.

13 $${\text{Q(}}\omega_{3} {) = }\sqrt {{\text{B}}_{s}^{2} + {\text{B}}_{c}^{2} } /T_{outq}$$

In the formula (13),

${\text{B}}_{s} = \frac{2}{mT}\int_{0}^{mT} {\overline{x}_{2} (t)\sin (\omega_{3} t)} dt$, ${\text{B}}_{{\text{c}}} = \frac{2}{mT}\int_{0}^{mT} {\overline{x}_{2} (t)\cos (\omega_{3} t)} dt$, m is a positive integer^20^.

**Figure 8:**
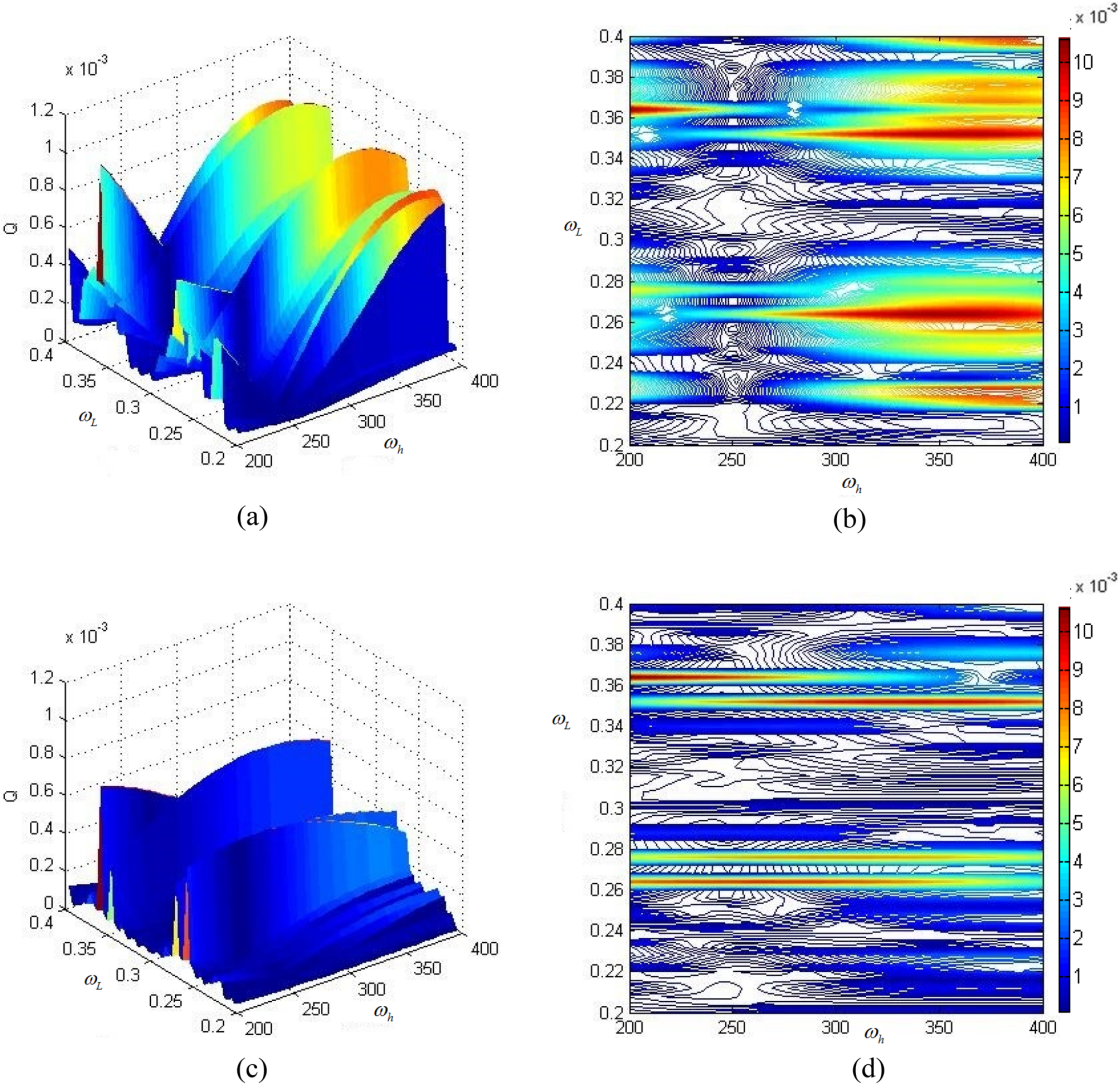
Influence of transmission system mass on amplitude-frequency characteristics under high and low frequency interference. (**a**) Influence of high and low frequency excitation frequencies on amplitude-frequency characteristics of common material transmission system under high and low frequency interference; (**b**) A projection corresponding to (**a**); (**c**) under the interference of high and low frequency, the influence of high and low frequency excitation frequencies and the amplitude-frequency characteristics of the corresponding box of braided mixed metal composite gear; (**d**) A projection corresponding to (**c**).

Figure 8a shows high and low frequency excitation frequencies under high and low frequency interference $\omega_{h}$ and $\omega_{L}$.Fig. 8b is (a) projection corresponding to Fig. 8a. Figure 8c shows the influence of high-frequency and low-frequency excitation frequencies on the amplitude-frequency characteristics of the braided mixed metal composite gear box, and Fig. 8d is a projection corresponding to Fig. 8c.

By comparing the four figures, it can be seen that with the decrease of the mass and moment of inertia of the transmission parts corresponding to the mixed metal composite gear, the amplitude-frequency characteristic Q of the common material box is larger than that of the woven mixed metal composite gear box. This conclusion shows that the decrease of the mass of the transmission parts has certain advantages in improving the dynamic characteristics of the system.

**Figure 9:**
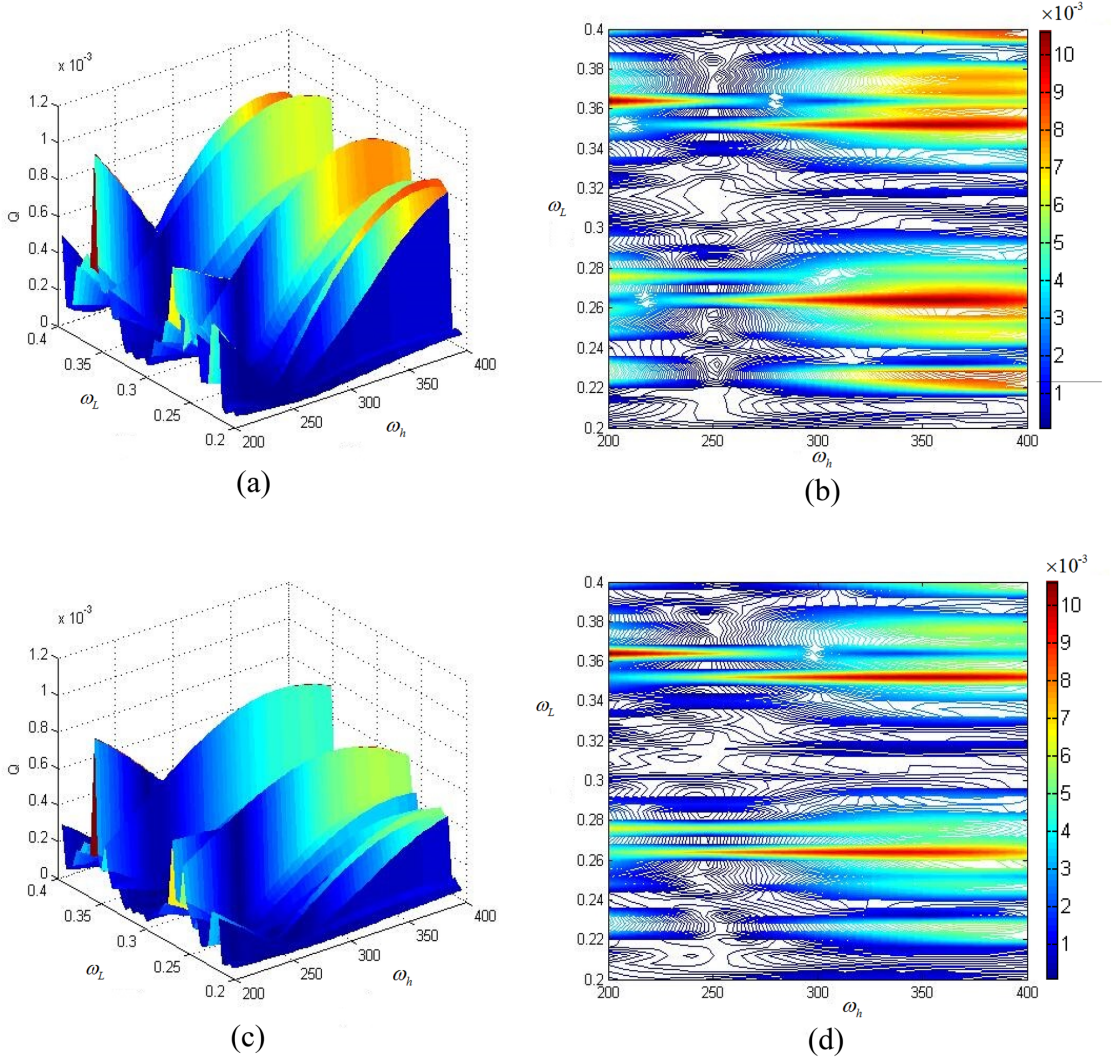
Influence of transmission system and box mass on amplitude-frequency characteristics under high and low frequency interference. (**a**) Ordinary gear and ordinary box; (**b**) A projection corresponding to (**a**); (**c**) lightweight gear and box; (**d**) A projection corresponding to (**c**).

Further, when the transmission gear is a braided mixed metal composite gear and the box is a braided composite box, under the interference of high and low frequencies, the excitation frequencies of high and low frequencies and the analysis results of the system are shown in Fig. 9. Figure 9a shows a common gear and a common box, Fig. 9b shows a projection corresponding to Fig. 9a, Fig. 9c shows a lightweight gear and box, and Fig. 9d shows a projection corresponding to Fig. 9c.

By comparing the four diagrams, it can be seen that the amplitude-frequency characteristic Q of lightweight gear and gearbox transmission system is slightly lower than that of ordinary gear and gearbox system, and the stability of the system is improved. This conclusion further shows that the lightweight design of two-stage gear transmission system can reduce the weight without sacrificing the dynamic characteristics of the system.

## Conclusion

In this paper, the braided composite material is applied to the reducer box and the gear web of mixed metal composite material. On this basis, the kinematic equations of the two-stage gear transmission system considering the box are established. By combining numerical and analytical methods, the influence of box mass, damping and connection mode on the dynamic characteristics of the composite two-stage gear reducer transmission system is studied. In addition, considering that the excitation of the box is complicated in the actual working process, this paper applies high and low frequency interference to the box respectively, and studies the dynamic characteristics of the system through vibration resonance analysis. The specific conclusions are as follows:

1. With the decrease of box mass, the overall trend of vibration displacement amplitude of high-speed shaft 1 decreases weakly, that of middle-speed shaft 2 decreases, that of low-speed shaft 4 decreases, and that of box 5 increases. Therefore, the decrease of box mass is beneficial to reduce the vibration of gear transmission parts.
2. Through the analytical analysis of the vibration displacement of the two-stage gear reducer and the box, it is found that the vibration displacement solution of the system consists of two parts: the steady-state response and the transient response, which correspond to the special solution of the non-homogeneous equation and the general solution of the homogeneous equation respectively. Because the natural frequencies of the composite components $\omega_{n}$ are higher than those of ordinary materials in different degrees, the $\varsigma \omega_{n}$ exponential term in the transient response solution increases, thus accelerating the decay process of the transient response, shortening the decay time and improving the dynamic characteristics of the system.
3. With the reduction of the mass and moment of inertia of the transmission parts corresponding to the mixed metal composite gear, the amplitude-frequency characteristic Q of the common material box is larger than that of the woven mixed metal composite gear box. Further research shows that the amplitude-frequency characteristic Q of the lightweight gear and gearbox transmission system is slightly lower than that of the common gear and gearbox system, and the stability of the system is increased, which shows that the influence of the transient response of the system on the composite transmission system is reduced in a certain frequency range under high and low frequency excitation.Therefore, the reduction of the mass of the lightweight transmission parts has certain advantages for improving the dynamic characteristics of the system (Supplementary Information).

### Supplementary Information

Supplementary Information 1.

Supplementary Information 2.

## Data Availability

All data generated or analysed during this study are included in this published article [and its supplementary information files].
