# Supplementary material for: Structural design and dynamic characteristics analysis of braided composite two-stage gear transmission system
Source: Sci Rep. 2024 Mar 7;14:5584. doi: 10.1038/s41598-024-56411-9 (PMC10920904; doi:10.1038/s41598-024-56411-9)
Supplement: Supplementary file 1 — Supplementary Information 1. [file 41598_2024_56411_MOESM1_ESM.docx]

**MATLAB program code of Fig.6**

**Time: December 28th, 2023**

clear all;clc;

v1=0.2;

v2=0.30;

E1=3e10;E2=1.2e11;

Ar=1e-6;%%%

Psi=0.89;%%%

H=45;%%%

Y=H/2.8;%%%%

gamma=1.5;

G=1.36E-9;

D=1.5;%%%%

k=0.454+0.41*v2;%%%%%

alpha=(2-D)/D*Ar;

E=1/((1-v1.^2)/E1+(1-v2.^2)/E2);

alphaC=(2.^(9-2*D)*pi.^(D-3)*G.^(2*D-2)*log(gamma)*((E/(k*H)).^2).^(1/(D-1)));

p1=1.38*2.8*(76.4.^(0.38)-1)*k*Y*D*Psi.^((2-D)/2)*alpha.^(D/2);

q1=1.14*(D-1)*2.^(4-D)*pi.^((D-2)/2)*(log(gamma)).^(1/2)*G.^(D-1);

p2=2*E*D*Psi.^((3-D)/2)*alpha.^(D/2)*(-alpha.^((1-D)/2)+alphaC.^((1-D)/2));

q2=(2*pi).^(1/2)*(1-D);

Kn=p1/q1+p2/q2;

Crj=1.0483e+003;

Krj=4.0000e+007;

% Crj=1.048e-2;

% Krj=3.19e-3;

fs=15;

T=2*pi;

t=0:T/fs:500*T;

N=length(t);

h=1/fs;%

pp=0.00000000*randn(size(t));

midu=7860;%%%Q235A

chi1=35;chi2=140;chi3=45;chi4=130;%%%%%

moshu=1.5;%%%%%%

r_1=moshu*chi1/2000;r_2=moshu*chi2/2000;r_3=moshu*chi3/2000;r_4=moshu*chi4/2000;

rn1=20/1000;rn2=20/1000;rn3=15/1000;rn4=15/1000;%

chihou1=30/1000;chihou2=30/1000;chihou3=25/1000;chihou4=25/1000;%%%%%%

J_1=(midu*pi*chihou1*((2*r_1).^4-(2*rn1).^4)/32);%kg。m^2

J_2=(midu*pi*chihou2*((2*r_2).^4-(2*rn2).^4)/32);%kg。m^2

J_3=(midu*pi*chihou3*((2*r_3).^4-(2*rn3).^4)/32);%kg。m^2

J_4=(midu*pi*chihou4*((2*r_4).^4-(2*rn4).^4)/32);%kg。m^2

J_23=J_2+J_3;

me1=(J_1*J_23)/(J_23*r_1.^2+J_1*r_2.^2);me2=J_23/(r_2*r_3);me3=J_4*J_23/(J_23*r_4.^2+J_4*r_3.^2);

y11=[];y22=[];

yali=cos(pi/9);bodong=1;aoufa=0;

m1=0.035;m2=1.34;m3=0.096;m23=m2+m3;m4=0.97;m5=5.69;m6=2.69;%%%

cxi_1=0.06;cxi_2=0.085;

%%%%%%%

%%%%%%%%%%%%%%%%%%%%

Cexi_b=10e+6;

cse1=100e-6;cse2=100e-6;cse3=100e-6;%%%%%%%%%%%%%%%%，

K1=10e-6;K2=10e-6;%%%%%%

%%%%%%%N/m，

%%%%%%%%%%%%%%%%%%%%

KK_1=2.32e+9;KK_3=1.79e+9;%%%%%

KK_15=5e+8;KK_25=5e+8;KK_45=5e+8;%%%%%

%%%%%%s/m，

%%%%%%%%%%%%%%%%%%%%

c1=2*cxi_1*sqrt(KK_1/(1/m1+1/m2));%%%%

c2=2*cxi_2*sqrt(KK_3/(1/m3+1/m4));%%%%

% c1=5500;c2=6857;

c15=1000;%%%

c45=1000;%%%

c25=1000;%%%

%Cn=4200;%%%%s/m，

Delta=0.65;%%%

Cn=Delta*((m1+m23+m4+m5)*Kn).^(1/2);%%%%%%%

w_h=(KK_1/me1).^(1/2);;%%%%%%

%%%%%%%m

%%%%%%%%%%%%%%%%%%%%

zhuansu=5000;%%r/min;

f1=zhuansu*chi1/60;f2=f1;

f3=zhuansu*chi1/chi2*chi3/60;f4=f3;

oumige1=2*pi*f1;oumige2=2*pi*f2;oumige3=2*pi*f3;oumige4=2*pi*f4;

Omega1=0.8;Omega2=Omega1;Omega3=0.2;Omega4=Omega3;%%%%

Omega15=0.6;Omega25=0.8;Omega45=0.4;

kk1=KK_1+KK_1*bodong*sin(Omega1.*t+aoufa);%%%%

kk2=KK_3+KK_3*bodong*sin(Omega3.*t+aoufa);%%%%

k15=KK_15+KK_15*bodong*sin(Omega15.*t+aoufa);%%%%

k25=(KK_1+KK_1*bodong*sin(Omega1.*t+aoufa)+KK_45+KK_3+KK_3*bodong*sin(Omega3.*t+aoufa))/2;%%%%

k45=KK_45+KK_45*bodong*sin(Omega45.*t+aoufa);%%%%

L=length(t);

y1=zeros(1,length(t)); z1=zeros(1,length(t)); %%%%%%

y2=zeros(1,length(t)); z2=zeros(1,length(t));

y4=zeros(1,length(t)); z4=zeros(1,length(t));

y5=zeros(1,length(t)); z5=zeros(1,length(t));

y6=zeros(1,length(t)); z6=zeros(1,length(t));

for i=1:(length(t)-1);

y1(i+1)=y1(i)*K1+h*z1(i)+pp(i);

z1(i+1)=z1(i)*K1+h*(yali*kk1(i)*K1/(w_h*w_h*m1)+c1*yali*(z1(i)-z2(i))/(w_h*m1)-c15*(z1(i)-z5(i))/(w_h*m1)-k15(i)*cse1/((w_h*w_h*m1))+(c15*(z1(i)-z5(i))+c25*(z2(i)-z5(i))+c45*(z4(i)-z5(i)))/(w_h*m5)+(k15(i)*cse1+k25(i)*cse2+k45(i)*cse3)/(w_h*w_h*m5)-Crj*(z5(i)-z6(i))/(w_h*m5)-Krj*(y5(i)-y6(i))/(w_h*w_h*m5));

y2(i+1)=y2(i)*K1+h*z2(i)+pp(i);

z2(i+1)=z2(i)*K1+h*(yali*kk1(i)*K1/(w_h*w_h*m23)+c1*yali*(z1(i)-z2(i))/(w_h*m23)-(yali*kk2(i)*K2)/(w_h*w_h*m23)-c2*yali*(z2(i)-z4(i))/(w_h*m23)-c25*(z2(i)-z5(i))/(w_h*m23)-k25(i)*cse2/(w_h*w_h*m23)+(c15*(z1(i)-z5(i))+c25*(z2(i)-z5(i))+c45*(z4(i)-z5(i)))/(w_h*m5)+(k15(i)*cse1+k25(i)*cse2+k45(i)*cse3)/(w_h*w_h*m5)-Crj*(z5(i)-z6(i))/(w_h*m5)-Krj*(y5(i)-y6(i))/(w_h*w_h*m5));

y4(i+1)=y4(i)*K1+h*z4(i)+pp(i);

z4(i+1)=z4(i)*K1+h*(yali*kk2(i)*K2/(w_h*w_h*m4)+c2*yali*(z2(i)-z4(i))/(w_h*m4)-c45*(z4(i)-z5(i))/(w_h*m4)-k45(i)*cse3/(w_h*w_h*m4)+(c15*(z1(i)-z5(i))+c25*(z2(i)-z5(i))+c45*(z4(i)-z5(i)))/(w_h*m5)+(k15(i)*cse1+k25(i)*cse2+k45(i)*cse3)/(w_h*w_h*m5)-Crj*(z5(i)-z6(i))/(w_h*m5)-Krj*(y5(i)-y6(i))/(w_h*w_h*m5));

y5(i+1)=y5(i)*K1+h*z5(i)+pp(i);

z5(i+1)=z1(i)*K1+h*((c15*(z1(i)-z5(i))+c25*(z2(i)-z5(i))+c45*(z4(i)-z5(i)))/(w_h*m5)+(k15(i)*cse1+k25(i)*cse2+k45(i)*cse3)/(w_h*w_h*m5)-Crj*(z5(i)-z6(i))/(w_h*m5)-Krj*(y5(i)-y6(i))/(w_h*w_h*m5));

y6(i+1)=y6(i)*K1+h*z6(i)+pp(i);

z6(i+1)=z6(i)*K1+h*((c15*(z1(i)-z5(i))+c25*(z2(i)-z5(i))+c45*(z4(i)-z5(i)))/(w_h*m5)+(k15(i)*cse1+k25(i)*cse2+k45(i)*cse3)/(w_h*w_h*m6)-Cn*z6(i)/(w_h*m5)-Kn*y6(i)/(w_h*w_h*m5));

end

gan=1e+8;

hold on;

subplot(5,2,1);

plot(t/w_h,y1,'r');

title('Displacement curve');

hold on;

hold on;

subplot(5,2,3);

plot(t/w_h,y2,'r');

title('Displacement curve');

hold on;

hold on;

subplot(5,2,5);

plot(t/w_h,y4,'r');

title('Displacement curve');

hold on;

hold on;

subplot(5,2,7);

plot(t/w_h,y5,'r');

title('Displacement curve');

hold on;

hold on;

subplot(5,2,9);

plot(t/w_h,y6,'r');

title('Displacement curve');

hold on;

%%%%%%%%%%%%%%%%%%%%%%%%%%%

%%%%%%%Kn、Cn%%%%%%%%%%%%%%*%%%%%

clear all;clc;

v1=0.2;%%%%

v2=0.30;%%%%%

E1=3e10;%%%%

E2=1.2e11;%%%N/m2

Ar=1e-6;%%%m2

Psi=0.89;%%%

H=45;%%%H15.778-56.35

Y=H/2.8;%%%%H

gamma=1.5;

G=1.36E-9;

D=1.5;%%%%1.1-1.9

k=0.454+0.41*v2;%%%%%

alpha=(2-D)/D*Ar;

E=1/((1-v1.^2)/E1+(1-v2.^2)/E2);

alphaC=(2.^(9-2*D)*pi.^(D-3)*G.^(2*D-2)*log(gamma)*((E/(k*H)).^2).^(1/(D-1)));

p1=1.38*2.8*(76.4.^(0.38)-1)*k*Y*D*Psi.^((2-D)/2)*alpha.^(D/2);

q1=1.14*(D-1)*2.^(4-D)*pi.^((D-2)/2)*(log(gamma)).^(1/2)*G.^(D-1);

p2=2*E*D*Psi.^((3-D)/2)*alpha.^(D/2)*(-alpha.^((1-D)/2)+alphaC.^((1-D)/2));

q2=(2*pi).^(1/2)*(1-D);

Kn=p1/q1+p2/q2;

Crj=1.0483e+003;

Krj=4.0000e+007;

% Crj=1.048e-2;

% Krj=3.19e-3;

%%%%%%%%%%%%%%%%%%%%%%%%%%%%%%%%%%%%%

%%%%%%%%%%%%%%%%%%%%%%%%%%%

%%%%%%%%%%%%%%%%%%%%%%%%%%%%%%%%%%%%%

fs=15;

T=2*pi;

t=0:T/fs:500*T;

N=length(t);

h=1/fs;%

pp=0.00000000*randn(size(t));

midu=7860;%%%Q235A，kg/m3

chi1=35;chi2=140;chi3=45;chi4=130;%%%%%

moshu=1.5;%%%%%%

r_1=moshu*chi1/2000;r_2=moshu*chi2/2000;r_3=moshu*chi3/2000;r_4=moshu*chi4/2000;%:m

rn1=20/1000;rn2=20/1000;rn3=15/1000;rn4=15/1000;%m

chihou1=30/1000;chihou2=30/1000;chihou3=25/1000;chihou4=25/1000;%%%%%%

J_1=0.5*(midu*pi*chihou1*((2*r_1).^4-(2*rn1).^4)/32);%kg。m^2

J_2=0.5*(midu*pi*chihou2*((2*r_2).^4-(2*rn2).^4)/32);%kg。m^2

J_3=0.5*(midu*pi*chihou3*((2*r_3).^4-(2*rn3).^4)/32);%kg。m^2

J_4=0.5*(midu*pi*chihou4*((2*r_4).^4-(2*rn4).^4)/32);%kg。m^2

J_23=J_2+J_3;

me1=(J_1*J_23)/(J_23*r_1.^2+J_1*r_2.^2);me2=J_23/(r_2*r_3);me3=J_4*J_23/(J_23*r_4.^2+J_4*r_3.^2);

y11=[];y22=[];

yali=cos(pi/9);bodong=1;aoufa=0;

%%%%%%%kg

%%%%%%%%%%%%%%%%%%%%

m1=0.5*0.035;m2=0.5*1.34;m3=0.5*0.096;m23=m2+m3;m4=0.5*0.97;m5=5.69;m6=2.69;%%%

%%%%%ok，0.07-0.1，

cxi_1=0.06;cxi_2=0.085;

%%%%%%%m

%%%%%%%%%%%%%%%%%%%%

Cexi_b=10e+6;

cse1=100e-6;cse2=100e-6;cse3=100e-6;%%%%%%%%%%%%%%%%

K1=10e-6;K2=10e-6;%%%%%%

%%%%%%%N/m，

%%%%%%%%%%%%%%%%%%%%

KK_1=2.32e+9;KK_3=1.79e+9;%%%%%

KK_15=5e+8;KK_25=5e+8;KK_45=5e+8;%%%%%

%%%%%%N。s/m，

%%%%%%%%%%%%%%%%%%%%

c1=2*cxi_1*sqrt(KK_1/(1/m1+1/m2));%%%%

c2=2*cxi_2*sqrt(KK_3/(1/m3+1/m4));%%%%

% c1=5500;c2=6857;

c15=1000;%%%

c45=1000;%%%

c25=1000;%%%

%Cn=4200;%%%%N。s/m

Delta=0.65;%%%

Cn=Delta*((m1+m23+m4+m5)*Kn).^(1/2);%%%%%%%

w_h=(KK_1/me1).^(1/2);;%%%%%%

%%%%%%%m

%%%%%%%%%%%%%%%%%%%%

zhuansu=5000;%%r/min;

f1=zhuansu*chi1/60;f2=f1;

f3=zhuansu*chi1/chi2*chi3/60;f4=f3;

oumige1=2*pi*f1;oumige2=2*pi*f2;oumige3=2*pi*f3;oumige4=2*pi*f4;

Omega1=0.8;Omega2=Omega1;Omega3=0.2;Omega4=Omega3;%%%%

Omega15=0.6;Omega25=0.8;Omega45=0.4;

kk1=KK_1+KK_1*bodong*sin(Omega1.*t+aoufa);%%%%

kk2=KK_3+KK_3*bodong*sin(Omega3.*t+aoufa);%%%%

k15=KK_15+KK_15*bodong*sin(Omega15.*t+aoufa);%%%%

k25=(KK_1+KK_1*bodong*sin(Omega1.*t+aoufa)+KK_45+KK_3+KK_3*bodong*sin(Omega3.*t+aoufa))/2;%%%%

k45=KK_45+KK_45*bodong*sin(Omega45.*t+aoufa);%%%%

L=length(t);

y1=zeros(1,length(t)); z1=zeros(1,length(t)); %%%%%%

y2=zeros(1,length(t)); z2=zeros(1,length(t));

y4=zeros(1,length(t)); z4=zeros(1,length(t));

y5=zeros(1,length(t)); z5=zeros(1,length(t));

y6=zeros(1,length(t)); z6=zeros(1,length(t));

for i=1:(length(t)-1);

y1(i+1)=y1(i)*K1+h*z1(i)+pp(i);

z1(i+1)=z1(i)*K1+h*(yali*kk1(i)*K1/(w_h*w_h*m1)+c1*yali*(z1(i)-z2(i))/(w_h*m1)-c15*(z1(i)-z5(i))/(w_h*m1)-k15(i)*cse1/((w_h*w_h*m1))+(c15*(z1(i)-z5(i))+c25*(z2(i)-z5(i))+c45*(z4(i)-z5(i)))/(w_h*m5)+(k15(i)*cse1+k25(i)*cse2+k45(i)*cse3)/(w_h*w_h*m5)-Crj*(z5(i)-z6(i))/(w_h*m5)-Krj*(y5(i)-y6(i))/(w_h*w_h*m5));

y2(i+1)=y2(i)*K1+h*z2(i)+pp(i);

z2(i+1)=z2(i)*K1+h*(yali*kk1(i)*K1/(w_h*w_h*m23)+c1*yali*(z1(i)-z2(i))/(w_h*m23)-(yali*kk2(i)*K2)/(w_h*w_h*m23)-c2*yali*(z2(i)-z4(i))/(w_h*m23)-c25*(z2(i)-z5(i))/(w_h*m23)-k25(i)*cse2/(w_h*w_h*m23)+(c15*(z1(i)-z5(i))+c25*(z2(i)-z5(i))+c45*(z4(i)-z5(i)))/(w_h*m5)+(k15(i)*cse1+k25(i)*cse2+k45(i)*cse3)/(w_h*w_h*m5)-Crj*(z5(i)-z6(i))/(w_h*m5)-Krj*(y5(i)-y6(i))/(w_h*w_h*m5));

y4(i+1)=y4(i)*K1+h*z4(i)+pp(i);

z4(i+1)=z4(i)*K1+h*(yali*kk2(i)*K2/(w_h*w_h*m4)+c2*yali*(z2(i)-z4(i))/(w_h*m4)-c45*(z4(i)-z5(i))/(w_h*m4)-k45(i)*cse3/(w_h*w_h*m4)+(c15*(z1(i)-z5(i))+c25*(z2(i)-z5(i))+c45*(z4(i)-z5(i)))/(w_h*m5)+(k15(i)*cse1+k25(i)*cse2+k45(i)*cse3)/(w_h*w_h*m5)-Crj*(z5(i)-z6(i))/(w_h*m5)-Krj*(y5(i)-y6(i))/(w_h*w_h*m5));

y5(i+1)=y5(i)*K1+h*z5(i)+pp(i);

z5(i+1)=z1(i)*K1+h*((c15*(z1(i)-z5(i))+c25*(z2(i)-z5(i))+c45*(z4(i)-z5(i)))/(w_h*m5)+(k15(i)*cse1+k25(i)*cse2+k45(i)*cse3)/(w_h*w_h*m5)-Crj*(z5(i)-z6(i))/(w_h*m5)-Krj*(y5(i)-y6(i))/(w_h*w_h*m5));

y6(i+1)=y6(i)*K1+h*z6(i)+pp(i);

z6(i+1)=z6(i)*K1+h*((c15*(z1(i)-z5(i))+c25*(z2(i)-z5(i))+c45*(z4(i)-z5(i)))/(w_h*m5)+(k15(i)*cse1+k25(i)*cse2+k45(i)*cse3)/(w_h*w_h*m6)-Cn*z6(i)/(w_h*m5)-Kn*y6(i)/(w_h*w_h*m5));

end

gan=1e+8;

hold on;

subplot(5,2,1);

plot(t/w_h,y1,'b');

title('Displacement curve');

hold on;

hold on;

subplot(5,2,3);

plot(t/w_h,y2,'b');

title('Displacement curve');

hold on;

hold on;

subplot(5,2,5);

plot(t/w_h,y4,'b');

title('Displacement curve');

hold on;

hold on;

subplot(5,2,7);

plot(t/w_h,y5,'b');

title('Displacement curve');

hold on;

hold on;

subplot(5,2,9);

plot(t/w_h,y6,'b');

title('Displacement curve');

hold on;
