# Supplementary material for: Structural design and dynamic characteristics analysis of braided composite two-stage gear transmission system
Source: Sci Rep. 2024 Mar 7;14:5584. doi: 10.1038/s41598-024-56411-9 (PMC10920904; doi:10.1038/s41598-024-56411-9)
Supplement: Supplementary file 2 — Supplementary Information 2. [file 41598_2024_56411_MOESM2_ESM.docx]

**MATLAB program code of Fig.8**

**Time: December 28th, 2023**

%%%%%%%%%%%%%%%%%%%%%%%%%%%%%

%%%%%%%%%Kn、Cn%%%%%%%%%%%%*%%%%%

clear all;clc;

v1=0.2;%%%%v1

v2=0.25;%%%%%v2

E1=3e10;%%%%N/m2

E2=1.3e11;%%%N/m2

Ar=1e-6;%%%Arm2

Psi=0.89;%%%

H=50;%%%H15.778-56.35

Y=H/2.8;%%%%H

gamma=1.5;

G=1.36E-9;

D=1.1;%%%%1.1-1.9

k=0.454+0.41*v2;%%%%%

alpha=(2-D)/D*Ar;

E=1/((1-v1.^2)/E1+(1-v2.^2)/E2);

alphaC=(2.^(9-2*D)*pi.^(D-3)*G.^(2*D-2)*log(gamma)*((E/(k*H)).^2).^(1/(D-1)));

p1=1.38*2.8*(76.4.^(0.38)-1)*k*Y*D*Psi.^((2-D)/2)*alpha.^(D/2);

q1=1.14*(D-1)*2.^(4-D)*pi.^((D-2)/2)*(log(gamma)).^(1/2)*G.^(D-1);

p2=2*E*D*Psi.^((3-D)/2)*alpha.^(D/2)*(-alpha.^((1-D)/2)+alphaC.^((1-D)/2));

q2=(2*pi).^(1/2)*(1-D);

Kn=p1/q1+p2/q2;

%%%%%%%%%%%%%%%%%%%%%%%%%%%%%%%%%%%%%

%%%%%%%%%%%%%%%%%%%%%%%%%%%%

%%%%%%%%%%%%%%%%%%%%%%%%%%%%%%%%%%%%%

N=51;%

fs=6.28/pi;

T=1/fs;%

t=(0:N-1)*T;

h=1/fs;%

gaopin=200:4:400;

dipin=0.2:0.004:0.4;

midu=7350;%%%kg/m3

chi1=35;chi2=140;chi3=45;chi4=130;%%%%%

moshu=1.5;%%%%%%

r_1=moshu*chi1/2000;r_2=moshu*chi2/2000;r_3=moshu*chi3/2000;r_4=moshu*chi4/2000;%:m

rn1=20/1000;rn2=20/1000;rn3=15/1000;rn4=15/1000;%m

chihou1=30/1000;chihou2=30/1000;chihou3=25/1000;chihou4=25/1000;%%%%%%

J_1=10*(midu*pi*chihou1*((2*r_1).^4-(2*rn1).^4)/32);%kg。m^2

J_2=10*(midu*pi*chihou2*((2*r_2).^4-(2*rn2).^4)/32);%kg。m^2

J_3=10*(midu*pi*chihou3*((2*r_3).^4-(2*rn3).^4)/32);%kg。m^2

J_4=10*(midu*pi*chihou4*((2*r_4).^4-(2*rn4).^4)/32);%kg。m^2

J_23=J_2+J_3;

me1=(J_1*J_23)/(J_23*r_1.^2+J_1*r_2.^2);

me2=J_23/(r_2*r_3);

me3=J_4*J_23/(J_23*r_4.^2+J_4*r_3.^2);

y11=[];y22=[];

yali=cos(pi/9);bodong=1;aoufa=0;yadao=(1/yali);

%%%%%%%kg

%%%%%%%%%%%%%%%%%%%%

m1=0.48;m2=1.5;m3=0.52;m23=m2+m3;m4=1.3;m5=22;;%%%

%%%%%ok，.07-0.1，

cxi_1=0.06;cxi_2=0.085;

%%%%%%%m

%%%%%%%%%%%%%%%%%%%%

Cexi_b=10e+6;

cse1(1)=200e-6;cse2(1)=180e-6;cse3(1)=160e-6;%%%%%%%%%%%%%%%%，K1(1)=140e-6;SiK2(1)=120e-6;%%%%%%

%%%%%%%N/m，

%%%%%%%%%%%%%%%%%%%%

KK_1=3e+8;KK_3=2e+8;%%%%%

KK_15=3.8e+8;KK_25=2.8e+8;KK_45=1.8e+8;%%%%%

%%%%%%N。s/m，

%%%%%%%%%%%%%%%%%%%%

c1=2*cxi_1*sqrt(KK_1/(1/m1+1/m2));%%%%

c2=2*cxi_2*sqrt(KK_3/(1/m3+1/m4));%%%%

% c1=5500;c2=6857;

c15=1000;%%%

c45=1000;%%%

c25=1000;%%%

%Cn=4200;%%%%N。s/m

Cn=0.04*((m1+m23+m4+m5)*Kn).^(1/2);%%%%%%%

w_h=(KK_1/me1).^(1/2);;%%%%%%

%%%%%%%m

%%%%%%%%%%%%%%%%%%%%

zhuansu=2000;%%r/min;

f1=zhuansu*chi1/60;f2=f1;

f3=zhuansu*chi1/chi2*chi3/60;f4=f3;

oumige1=2*pi*f1;oumige2=2*pi*f2;oumige3=2*pi*f3;oumige4=2*pi*f4;

Omega1=oumige1/w_h;Omega2=oumige2/w_h;Omega3=oumige3/w_h;Omega4=oumige4/w_h;

Omega15=0.4;%%%*****

Omega25=0.3;%%%----

Omega45=0.2;%%%

L=length(t);

J=length(gaopin);

S=length(dipin);

for r=1:J

for j=1:S

y1=zeros(1,length(t)); z1=zeros(1,length(t)); %%%%%%

y2=zeros(1,length(t)); z2=zeros(1,length(t));

y4=zeros(1,length(t)); z4=zeros(1,length(t));

y5=zeros(1,length(t)); z5=zeros(1,length(t));

for i=1:(length(t)-1);

pp(j,r)=0.01*(1e-8)*sin(dipin(j).*t(i)+aoufa)+500*(1e-8)*sin(gaopin(r).*t(i)+aoufa);

kk1=KK_1+KK_1*bodong*sin(Omega1.*t+aoufa);%%%%

kk2=KK_3+KK_3*bodong*sin(Omega3.*t+aoufa);%%%%

k15=KK_15+KK_15*bodong*sin(Omega15.*t+aoufa);%%%%

k25=KK_25+KK_25*bodong*sin(Omega25.*t+aoufa);%%%%

k45=KK_45+KK_45*bodong*sin(Omega45.*t+aoufa);%%%%

cse1=(y1(i)-y5(i)-cse1);

cse2=(y2(i)-y5(i)-cse2);

cse3=(y4(i)-y5(i)-cse3);

K1=(y1(i)-y2(i))*yadao+K1;

SiK2=(y4(i)-y2(i))*yadao+SiK2;

y1(i+1)=y1(i)/Cexi_b+h*z1(i)/Cexi_b;

z1(i+1)=z1(i)/Cexi_b+h*(yali*kk1(i)*K1/(w_h*w_h*m1)+c1*yali*(z1(i)-z2(i))/(w_h*m1)-c15*(z1(i)-z5(i))/(w_h*m1)-k15(i)*cse1/((w_h*w_h*m1))+(c15*(z1(i)-z5(i))+c25*(z2(i)-z5(i))+c45*(z4(i)-z5(i)))/(w_h*m5)+(k15(i)*cse1+k25(i)*cse2+k45(i)*cse3)/(w_h*w_h*m5)-Cn/(w_h*m5)*z5(i)-Kn/(w_h*w_h*m5).*y5(i));

y2(i+1)=y2(i)/Cexi_b+h*z2(i)/Cexi_b;

z2(i+1)=z2(i)/Cexi_b+h*(yali*kk1(i)*K1/(w_h*w_h*m23)+c1*yali*(z1(i)-z2(i))/(w_h*m23)-(yali*kk2(i)*SiK2)/(w_h*w_h*m23)-c2*yali*(z2(i)-z4(i))/(w_h*m23)-c25*(z2(i)-z5(i))/(w_h*m23)-k25(i)*cse2/(w_h*w_h*m23)+(c15*(z1(i)-z5(i))+c25*(z2(i)-z5(i))+c45*(z4(i)-z5(i)))/(w_h*m5)+(k15(i)*cse1+k25(i)*cse2+k45(i)*cse3)/(w_h*w_h*m5)-Cn/(w_h*m5)*z5(i)-Kn/(w_h*w_h*m5).*y5(i));

y4(i+1)=y4(i)/Cexi_b+h*z4(i)/Cexi_b;

z4(i+1)=z4(i)/Cexi_b+h*(yali*kk2(i)*SiK2/(w_h*w_h*m4)+c2*yali*(z2(i)-z4(i))/(w_h*m4)-c45*(z4(i)-z5(i))/(w_h*m4)-k45(i)*cse3/(w_h*w_h*m4)+(c15*(z1(i)-z5(i))+c25*(z2(i)-z5(i))+c45*(z4(i)-z5(i)))/(w_h*m5)+(k15(i)*cse1+k25(i)*cse2+k45(i)*cse3)/(w_h*w_h*m5)-Cn/(w_h*m5)*z5(i)-Kn/(w_h*w_h*m5).*y5(i));

y5(i+1)=y5(i)/Cexi_b+h*z5(i)/Cexi_b+pp(j,r);

z5(i+1)=z1(i)/Cexi_b+h*((c15*(z1(i)-z5(i))+c25*(z2(i)-z5(i))+c45*(z4(i)-z5(i)))/(w_h*m5)+(k15(i)*cse1+k25(i)*cse2+k45(i)*cse3)/(w_h*w_h*m5)-Cn/(w_h*m5)*z5(i)-Kn/(w_h*w_h*m5).*y5(i))+pp(j,r);

end

y11=z5.*sin(dipin(j)*t)*h;

y22=z5.*cos(dipin(j).*t)*h;

B1=sum(y11);B2=sum(y22);

Q=2/0.2*sqrt(B1^2+B2^2);

B(r,j)=Q;

end

end

subplot(2,2,1)

surf(gaopin(2:length(gaopin)),dipin(2:length(dipin)),B(2:length(t),2:length(t)))

xlabel('gaopin');ylabel('dipin');zlabel('Q');

subplot(2,2,2);

contour(gaopin,dipin,B,100)

xlabel('gaopin');ylabel('dipin');zlabel('Q');

%%%%%%%%%%%%%%%%%%%

%%%%%%%%%Kn、Cn%%%%%%%%%%%%%%*%%%%%

clear all;clc;

v1=0.2;%%%%v1

v2=0.25;%%%%%v2

E1=3e10;%%%%N/m2

E2=1.3e11;%%%N/m2

Ar=1e-6;%%%Arm2

Psi=0.89;%%%

H=50;%%%H15.778-56.35

Y=H/2.8;%%%%H

gamma=1.5;

G=1.36E-9;

D=1.1;%%%%1.1-1.9

k=0.454+0.41*v2;%%%%%

alpha=(2-D)/D*Ar;

E=1/((1-v1.^2)/E1+(1-v2.^2)/E2);

alphaC=(2.^(9-2*D)*pi.^(D-3)*G.^(2*D-2)*log(gamma)*((E/(k*H)).^2).^(1/(D-1)));

p1=1.38*2.8*(76.4.^(0.38)-1)*k*Y*D*Psi.^((2-D)/2)*alpha.^(D/2);

q1=1.14*(D-1)*2.^(4-D)*pi.^((D-2)/2)*(log(gamma)).^(1/2)*G.^(D-1);

p2=2*E*D*Psi.^((3-D)/2)*alpha.^(D/2)*(-alpha.^((1-D)/2)+alphaC.^((1-D)/2));

q2=(2*pi).^(1/2)*(1-D);

Kn=p1/q1+p2/q2;

%%%%%%%%%%%%%%%%%%%%%%%%%%%%%%%%%%%%%

%%%%%%%%%%%%%%%%%%%%%%%%%%%%%

%%%%%%%%%%%%%%%%%%%%%%%%%%%%%%%%%%%%%

N=51;%

fs=6.28/pi;

T=1/fs;%

t=(0:N-1)*T;

h=1/fs;%

gaopin=200:4:400;

dipin=0.2:0.004:0.4;

midu=7350;%%%kg/m3

chi1=35;chi2=140;chi3=45;chi4=130;%%%%%

moshu=1.5;%%%%%%

r_1=moshu*chi1/2000;r_2=moshu*chi2/2000;r_3=moshu*chi3/2000;r_4=moshu*chi4/2000;%:m

rn1=20/1000;rn2=20/1000;rn3=15/1000;rn4=15/1000;%:m

chihou1=30/1000;chihou2=30/1000;chihou3=25/1000;chihou4=25/1000;%%%%%%

J_1=2*(midu*pi*chihou1*((2*r_1).^4-(2*rn1).^4)/32);%kg。m^2

J_2=2*(midu*pi*chihou2*((2*r_2).^4-(2*rn2).^4)/32);%kg。m^2

J_3=2*(midu*pi*chihou3*((2*r_3).^4-(2*rn3).^4)/32);%kg。m^2

J_4=2*(midu*pi*chihou4*((2*r_4).^4-(2*rn4).^4)/32);%kg。m^2

J_23=J_2+J_3;

me1=(J_1*J_23)/(J_23*r_1.^2+J_1*r_2.^2);

me2=J_23/(r_2*r_3);

me3=J_4*J_23/(J_23*r_4.^2+J_4*r_3.^2);

y11=[];y22=[];

yali=cos(pi/9);bodong=1;aoufa=0;yadao=(1/yali);

%%%%%%%kg

%%%%%%%%%%%%%%%%%%%%

m1=0.28;m2=0.8;m3=0.22;m23=m2+m3;m4=0.45;m5=22;;%%%

%%%%%ok，0.07-0.1

cxi_1=0.06;cxi_2=0.085;

%%%%%%m

%%%%%%%%%%%%%%%%%%%%

Cexi_b=10e+6;

cse1(1)=200e-6;cse2(1)=180e-6;cse3(1)=160e-6;%%%%%%%%%%%%%%%%，K1(1)=140e-6;SiK2(1)=120e-6;%%%%%%

%%%%%%%N/m，

%%%%%%%%%%%%%%%%%%%%

KK_1=3e+8;KK_3=2e+8;%%%%%2

KK_15=3.8e+8;KK_25=2.8e+8;KK_45=1.8e+8;%%%%%

%%%%%%N。s/m，

%%%%%%%%%%%%%%%%%%%%

c1=2*cxi_1*sqrt(KK_1/(1/m1+1/m2));%%%%

c2=2*cxi_2*sqrt(KK_3/(1/m3+1/m4));%%%%

% c1=5500;c2=6857;

c15=1000;%%%

c45=1000;%%%

c25=1000;%%%

%Cn=4200;%%%%N。s/m

Cn=0.04*((m1+m23+m4+m5)*Kn).^(1/2);%%%%%%%

w_h=(KK_1/me1).^(1/2);;%%%%%%

%%%%%%%m

%%%%%%%%%%%%%%%%%%%%

zhuansu=2000;%r/min;

f1=zhuansu*chi1/60;f2=f1;

f3=zhuansu*chi1/chi2*chi3/60;f4=f3;

oumige1=2*pi*f1;oumige2=2*pi*f2;oumige3=2*pi*f3;oumige4=2*pi*f4;

Omega1=oumige1/w_h;Omega2=oumige2/w_h;Omega3=oumige3/w_h;Omega4=oumige4/w_h;

Omega15=0.4;%%%

Omega25=0.3;%%%

Omega45=0.2;%%%

L=length(t);

J=length(gaopin);

S=length(dipin);

for r=1:J

for j=1:S

y1=zeros(1,length(t)); z1=zeros(1,length(t)); %%%%%%

y2=zeros(1,length(t)); z2=zeros(1,length(t));

y4=zeros(1,length(t)); z4=zeros(1,length(t));

y5=zeros(1,length(t)); z5=zeros(1,length(t));

for i=1:(length(t)-1);

pp(j,r)=0.01*(1e-8)*sin(dipin(j).*t(i)+aoufa)+500*(1e-8)*sin(gaopin(r).*t(i)+aoufa);

kk1=KK_1+KK_1*bodong*sin(Omega1.*t+aoufa);%%%%

kk2=KK_3+KK_3*bodong*sin(Omega3.*t+aoufa);%%%%

k15=KK_15+KK_15*bodong*sin(Omega15.*t+aoufa);%%%%

k25=KK_25+KK_25*bodong*sin(Omega25.*t+aoufa);%%%% k45=KK_45+KK_45*bodong*sin(Omega45.*t+aoufa);%%%%

cse1=(y1(i)-y5(i)-cse1);

cse2=(y2(i)-y5(i)-cse2);

cse3=(y4(i)-y5(i)-cse3);

K1=(y1(i)-y2(i))*yadao+K1;

SiK2=(y4(i)-y2(i))*yadao+SiK2;

y1(i+1)=y1(i)/Cexi_b+h*z1(i)/Cexi_b;

z1(i+1)=z1(i)/Cexi_b+h*(yali*kk1(i)*K1/(w_h*w_h*m1)+c1*yali*(z1(i)-z2(i))/(w_h*m1)-c15*(z1(i)-z5(i))/(w_h*m1)-k15(i)*cse1/((w_h*w_h*m1))+(c15*(z1(i)-z5(i))+c25*(z2(i)-z5(i))+c45*(z4(i)-z5(i)))/(w_h*m5)+(k15(i)*cse1+k25(i)*cse2+k45(i)*cse3)/(w_h*w_h*m5)-Cn/(w_h*m5)*z5(i)-Kn/(w_h*w_h*m5).*y5(i));

y2(i+1)=y2(i)/Cexi_b+h*z2(i)/Cexi_b;

z2(i+1)=z2(i)/Cexi_b+h*(yali*kk1(i)*K1/(w_h*w_h*m23)+c1*yali*(z1(i)-z2(i))/(w_h*m23)-(yali*kk2(i)*SiK2)/(w_h*w_h*m23)-c2*yali*(z2(i)-z4(i))/(w_h*m23)-c25*(z2(i)-z5(i))/(w_h*m23)-k25(i)*cse2/(w_h*w_h*m23)+(c15*(z1(i)-z5(i))+c25*(z2(i)-z5(i))+c45*(z4(i)-z5(i)))/(w_h*m5)+(k15(i)*cse1+k25(i)*cse2+k45(i)*cse3)/(w_h*w_h*m5)-Cn/(w_h*m5)*z5(i)-Kn/(w_h*w_h*m5).*y5(i));

y4(i+1)=y4(i)/Cexi_b+h*z4(i)/Cexi_b;

z4(i+1)=z4(i)/Cexi_b+h*(yali*kk2(i)*SiK2/(w_h*w_h*m4)+c2*yali*(z2(i)-z4(i))/(w_h*m4)-c45*(z4(i)-z5(i))/(w_h*m4)-k45(i)*cse3/(w_h*w_h*m4)+(c15*(z1(i)-z5(i))+c25*(z2(i)-z5(i))+c45*(z4(i)-z5(i)))/(w_h*m5)+(k15(i)*cse1+k25(i)*cse2+k45(i)*cse3)/(w_h*w_h*m5)-Cn/(w_h*m5)*z5(i)-Kn/(w_h*w_h*m5).*y5(i));

y5(i+1)=y5(i)/Cexi_b+h*z5(i)/Cexi_b+pp(j,r);

z5(i+1)=z1(i)/Cexi_b+h*((c15*(z1(i)-z5(i))+c25*(z2(i)-z5(i))+c45*(z4(i)-z5(i)))/(w_h*m5)+(k15(i)*cse1+k25(i)*cse2+k45(i)*cse3)/(w_h*w_h*m5)-Cn/(w_h*m5)*z5(i)-Kn/(w_h*w_h*m5).*y5(i))+pp(j,r);

end

y11=z5.*sin(dipin(j)*t)*h;

y22=z5.*cos(dipin(j).*t)*h;

B1=sum(y11);B2=sum(y22);

Q=2/0.2*sqrt(B1^2+B2^2);

B(r,j)=Q;

end

end

subplot(2,2,3)

surf(gaopin(2:length(gaopin)),dipin(2:length(dipin)),B(2:length(t),2:length(t)))

xlabel('gaopin');ylabel('dipin');zlabel('Q');

subplot(2,2,4);

contour(gaopin,dipin,B,100)

xlabel('gaopin');ylabel('dipin');zlabel('Q');
